# Small molecule organic eutectics as candidates to replace plastics†

**DOI:** 10.1039/d4sc02574a

**Published:** 2024-08-12

**Authors:** Joshua L. Ryan, Gabriele C. Sosso, Stefan A. F. Bon

**Affiliations:** a Department of Chemistry, The University of Warwick Coventry CV4 7AL UK https://bonlab.info s.bon@warwick.ac.uk g.sosso@warwick.ac.uk

## Abstract

Legislative change and shifting consumer sentiment drive a need to replace polymers in certain products. Herein, we highlight that eutectic molecular glasses and liquids are promising but underutilized candidate materials. We formulate a series of hydrophobic eutectic molecular liquids and glasses by mixing their crystalline components. The eutectic composition of each mixture was determined by both differential scanning calorimetry (DSC) and UV-vis spectroscopic measurements, which were processed and analyzed using a trained partial least squares regression model. With product shelf-life in mind, the long-term stability (up to 14 months) of the amorphous materials towards crystallization was proven using powder X-ray diffraction (PXRD). Molecular dynamics (MD) simulations put forward potential design rules in terms of the physical stability of these glasses. Rheological properties were investigated from the perspective of processability. Low fragility indices were found for all liquids, aiding processability through glassblowing, fiber pulling, film formation and molding. We show that properties can be tailored by blending two different eutectic systems or simply adding a plasticizer. To demonstrate a potential application area, the 4-hydroxychalcone and bifonazole eutectic system was used as a matrix for controlled release studies of a model active ingredient.

## Abstract 2

Mixing small organic crystalline molecules produces eutectic glasses and liquids with “polymer-like” physical properties.

## Graphical abstract

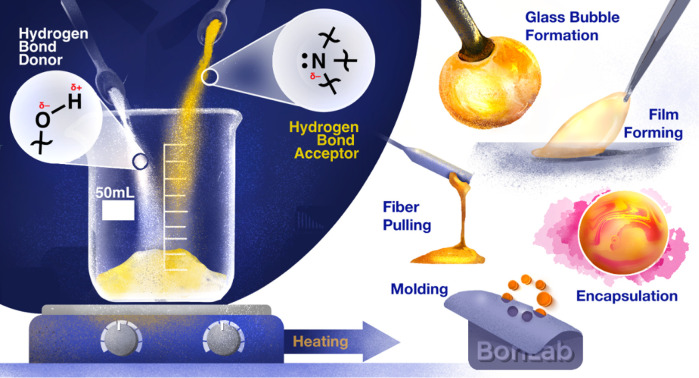

## Introduction

A variety of glassy, and thus amorphous, materials have been used by humanity throughout history and into the modern day. This is a direct result of their unique properties and versatility. Different types of glasses exist and can be distinguished by their molecular or colloidal building blocks.^1–4^ The natural glass, obsidian, was first used thousands of years ago to make jewelry and weapons.^5^ Trehalose, in its glassy state, plays a critical role in the self-preservation of insect life in extreme climates.^6,7^ The deep sea cables driving the internet rely on optical fibers containing amorphous silica.^8^

One of the more underutilized variants are organic glasses composed of small molecules. These amorphous solids are typically formed through processes such as melt-quenching, solvent evaporation, or vapor deposition.^9,10^ A well-known molecular organic glass is sugar glass, made from cooling a hot (150 °C) aqueous sucrose solution in the presence of glucose or corn syrup. This is used in confectionery, sculptures, and movie props.^11–14^ Recent years have seen applications of small-molecule organic glasses expand significantly, particularly in the organic electronics industry. Here organic glasses are enabling innovation in the form of organic light-emitting diode (OLED) displays, a common feature of current high-end TVs. Triarylamine-based molecular glasses are used in the amorphous hole-transport layers of such devices.^15,16^ Another example is the use of molecular glasses as photoresists in nanolithography.^11^ Compared to traditionally used materials, molecular glasses form smoother patterns with higher resolution. A shared feature of small-molecule organic glasses in OLEDs and nanolithography is they replace polymers in these applications.

We posed the question of whether the design of small-molecule glasses could be tailored to feature desirable physical properties and characteristics so they ultimately can replace polymers in a wide range of applications. Polymeric materials have undoubtedly transformed and enriched our lives due to their versatility and desirable properties. One exceptional and at the same time not-so exceptional property possessed by many polymers is their durability and thus inability to degrade. It is well-known that the accumulation of plastic in the environment is a problem. For example, 45% of marine mammal species are at risk of harm through entanglement or ingestion of plastic.^17,18^ The exposure of marine corals to high concentrations of poly(ethylene) microparticles has been shown to illicit bleaching and necrosis.^19^ The adverse environmental impacts are driving enhanced regulatory pressure against, and shifts in consumer purchasing away from, polymer-containing products. The ECHA proposes to ban products containing non-degrading, intentionally added microplastics in almost all applications following specific transition periods of 4–12 years.^20^ The result is an urgent rethink on how to formulate and design products and replace polymers with other materials that are less problematic at the product's end-of-life. Our focus is on opportunities to replace synthetic polymers in the first instance in formulations designed for coatings and adhesives, and for drug and beneficial agent delivery systems. The idea is to start exploring what small-molecule glasses can offer and highlight what they can provide regarding some of their physical characteristics from a polymer perspective.

In pursuit of this challenge, we looked at a subset of small-molecule organic mixtures that form deep eutectic solvents (DES).^21^ The formation of organic DES typically involves mixing certain hydrogen bond donor–acceptor pairs in a specific molar ratio under heat. Upon cooling, the desired result is a liquid. DES are prone to supercooling and glass formation.^22^ Due to their intended application as solvents, low viscosity DES are often selectively pursued as these exhibit favorable processibility and transport properties. Viscous or glass-forming DES are discarded and consequently understudied.

Herein would like to show that strikingly these viscous eutectic liquids and glasses are interesting candidates to replace certain thermoplastic polymer systems. We are not saying that these materials at this stage can replace commodity and engineering plastics. We believe that small molecule organic glasses deserve a closer look, and hope this paper will trigger the enthusiasm to develop this area. We would like to point out an excellent recent study by Yan and co-workers, who reported single-molecule melt-quenched glasses as plastic alternatives.^23^ We believe our approach adds versatility as a platform in that glass transition temperatures and viscosities of the liquids can be tuned.

We first screened binary mixtures of low molecular weight hydrophobic crystalline donor–acceptor pairs for both eutectic and glass-forming behavior. Two approaches were taken for eutectic composition determination – the first being Differential Scanning Calorimetry (DSC) and the second UV-vis coupled with supervised machine learning in the form of partial least squares regression (PLSR). The stability of the resultant eutectic molecular glasses towards crystallization was then followed over many months using X-ray diffraction (XRD). Molecular dynamics (MD) simulations offered atomistic insight into the physical stability of different mixtures. Processability is highlighted briefly in the form of glassblowing, molding, fiber pulling and thin film formation. Rheological measurements then probed each liquid for non-Newtonian behavior and proved the tunable nature of their physical properties. Finally, the utility of these eutectic molecular glasses as encapsulation matrices was demonstrated by tracking diffusion kinetics as a function of temperature.

## Experimental

### Materials

All solvents and reagents were used as received without further purification. 4-Hydroxychalcone (97%), 4′-hydroxychalcone (97%), bifonazole (98%), rosolic acid (95%), phloroglucide (95%), nicotinamide (99%), curcumin (95%) and rhodamine WT (20% solution in water, isolated prior to use) were purchased from Thermo Scientific. Lavandin oil, tannic acid, *trans*-chalcone (97%) and dimethylsulfoxide-d_6_ (99%) were purchased from Sigma Aldrich. Climbazole (97%) was purchased from Yancheng Lvye. Differential scanning calorimetry was performed using a TA Instruments DSC2500 with 40 μL hermetically sealed aluminium pans, purchased from Thermal Instruments Ltd. The instrument was calibrated with indium and operated with a nitrogen flow rate of 50 mL min^−1^. A temperature ramp rate of 10 °C min^−1^ was used in all cases. Rheology measurements were performed on a TA Instruments DHR-3 rheometer with upper (8 mm) and lower (50 mm) Peltier temperature control in parallel plate geometry. UV-vis absorbance spectroscopy was conducted using an Agilent Cary 60 or Cary 3500 spectrophotometer measuring between 200–800 nm in 1 nm steps. An airtight Hellma Analytics quartz cuvette (117100-F-10-40) with PTFE-coated silicone seals and a path length of 10 mm was used for all measurements. Powder X-ray diffraction measurements were performed at 25 °C using a Panalytical Empyrean Alpha-1 Diffractometer with an unmonochromated Cu source (Kα_1_, *λ* = 1.54056 Å and Kα_2_, *λ* = 1.54443 Å) operating in reflection mode with a PIXcel3D detector.

### Differential scanning calorimetry (DSC)

Each binary mixture was prepared in 10 mol% steps and transferred to aluminum pans wherein they were hermetically sealed. The reference pan was left empty and sealed the same way. The reference and sample were heated from RT to a temperature below the melting point (*T*_m_) of the lowest melting compound and held isothermally for 20–30 min to allow formation of the eutectic melt phase. The temperature was then decreased far below the glass transition temperature (*T*_g_) and held isothermally for 5–30 min. Two heat-cool cycles followed (with intermediate isotherm steps), heating above *T*_m_ of both compounds to melt excess crystalline material, before cooling again far below *T*_g_. The experiment was repeated using a more granular, 2 mol% step around the eutectic composition once a rough position was determined from initial tests. See Fig. S1† for exact details.

### Tammann diagram construction

The eutectic and excess melting peaks obtained *via* DSC were integrated to obtain their respective heats of fusion (*H*_fus_). *H*_fus_*vs.* composition was visualized as a scatter plot and linear regression applied in four separate regions. The regions of increasing and decreasing *H*_fus_ were each fitted separately for both the eutectic and excess melt data. The regression lines for each overlap, producing a peak in the eutectic and a trough in the excess melt data. The position of these along the composition axis gives the eutectic composition, taken as an average of the two values.

### Partial least squares regression (PLSR) analysis of UV-vis data

PLSR is a supervised machine learning technique which finds the relationship between dependent and independent variables.^24^ The concentration and full absorbance (200–800 nm) spectrum of Cha/Bif calibration samples were used as dependent and independent variables respectively to form the basis of the model. The data was split into a training set (40 samples), used to develop the model, and a test set (10 samples), used to obtain a preliminary measure of model accuracy in terms of *R*^2^ between predicted and actual Cha/Bif concentrations. Regression was then performed on the training set, predicting values of the dependent variables using the decomposition of the independent variables. The optimal number of latent variables (LVs) was taken as that which yields the lowest root mean square error of cross-validation (RMSECV, Fig. S2†). LVs are random variables extracted from the training set which reduce the dimensionality of the dataset while still explaining as much of the covariance as possible. The performance of the model was further evaluated using 5-fold cross-validation. Cross-validation is especially suitable for small to medium-sized datasets, since partitioning can introduce significant bias.^24^ All chemometric analysis was carried out in Python using the packages: Scikit-learn, Numpy, Pandas and Matplotlib. The Python code is available on Github (https://doi.org/10.5281/zenodo.8157643).

### UV-vis sample preparation

Stock solutions were prepared by dissolution of Cha and/or Bif, or RhWT for release experiments, in EtOH. An appropriate mass of each stock solution was diluted further with EtOH to achieve the desired concentration. Each sample is derived from a unique stock solution to eliminate the propagation of any errors arising from sample preparation. For example, Cha (10.0 mg) and Bif (31.5 mg) were dissolved in EtOH (100 mL) to produce a stock solution of 0.10 mg mL^−1^ Cha and 0.32 mg mL^−1^ Bif. An aliquot of this stock was diluted 78.3-fold (0.1008 g in a 10 mL volumetric flask), producing a sample of 1.6 μg mL^−1^ Cha and 4.0 μg mL^−1^ Bif. See Table S1† for details.

### UV-vis absorbance measurements

Samples of the desired Cha and Bif concentration (see above) were transferred to an airtight quartz cuvette and their absorbance measured between 200–800 nm. An airtight cuvette was used to eliminate concentration increases due to EtOH evaporation. All absorbance spectra were baseline subtracted against EtOH.

### Preparation of molecular glasses

Compounds were combined as binary mixtures at their respective eutectic composition. Each mixture was stirred and heated at its eutectic melt temperature (*T*_eu_) until a homogenous liquid was formed, before cooling to room temperature. With Cha/Bif as an example, 4-hydroxychalcone (34.3 mg, 0.16 mmol) and bifonazole (65.7 mg, 0.21 mmol) were added to a vial, premixed, heated at 116 °C and stirred for 1 h before cooling to room temperature, see ESI Video.† The materials produced were used directly in later tests. See Table S2† for exact details.

### Amorphous phase stability

Samples were transferred into a Si holder as either a fine powder, ground with a pestle and mortar, or as a viscous liquid. Spectra were recorded in the 2*θ* range 5–75° (step size 0.013°) with each experiment totaling 30 or 60 min. The diffraction pattern of each sample was measured once per week for 4 or 5 weeks and monthly thereafter to probe for crystal growth. Diffraction patterns were normalized to enable comparisons to be made more easily.

### Viscosity-temperature dependence

Samples were loaded in the molten state, at 120 °C, and conditioned for 300 s. A cool–heat cycle was then initiated over the relevant temperature range, ramping at a rate of 2 °C min^−1^ and under constant velocity of 1.0 rad s^−1^. See Table S3† for further details.

### Linear viscoelastic region (LVER) determination

The linear viscoelastic region (LVER) of each material was defined using an amplitude sweep. Samples were isothermally conditioned for 300 s, before initiation of an oscillation amplitude sweep from 2.0 × 10^−8^ to 10.0 rad at angular frequency of 5.0 or 10.0 rad s^−1^. See Table S4† for further details.

### Viscosity-shear rate dependence

Shear-dependent behavior was probed using frequency sweeps. Samples were isothermally conditioned for 300 s, before initiation of an oscillation frequency sweep from 0.1 to 100 rad s^−1^ with displacement chosen as being within the LVER as per the prior amplitude sweep measurements. The experiment was repeated over a range of temperatures. See Table S5† for further details.

### Viscoelastic characteristics

Oscillatory temperature sweeps were performed to examine the viscoelastic properties of each sample. Samples were isothermally conditioned for 300 s, before initiation of a cool–heat cycle at a rate of 2 °C min^−1^ with constant angular frequency and displacement. See Table S6† for further details.

### Plasticization experiments

Texanol was added to the relevant glass in varying quantities (1, 5, 10, 25 wt%) before heating to 100 °C and stirring until homogenous. Oscillatory temperature sweeps were then performed as outlined above and in Table S6.†

### Encapsulate release measurements

A Cha/Bif glass film (100 mg, dimensions = 10 × 10 × 1.2 mm) containing 39.5 μg rhodamine WT (*λ*_max_ = 556 nm) was cast on the inner base of a quartz cuvette. Deionized water (2 mL) was added, and the absorbance of the liquid measured between 200–800 nm every 1 min for at least 10 hours until a constant value was reached. Absorbance was converted to concentration using a calibration plot of known standards (Fig. S3†).

### Molecular dynamics (MD) simulations

We have chosen to focus on two binary mixtures: (1) curcumin/bifonazole (Cur/Bif, *χ*_Cur_ = 0.35), and; (2) curcumin/climbazole (Cur/Cli, *χ*_Cur_ = 0.22). In both cases, we have considered 512 molecules (Cur = 179 and Bif = 333 for Cur/Bif and Cur = 113 and Cli = 399 for Cur/Cli). The edge of the (cubic) simulation boxes ranged from ∼7 to 6 nm (according to the temperature conditions, see below). The initial configurations were prepared *via* Packmol.^25^ CHARMM-GUI was used to generate the topologies of curcumin, bifonazole and climbazole *via* the CGenFF force field.^26,27^ The resulting penalties in terms of the force field parameters (charges included) were between 25 and 55. Whilst these are clearly sub-optimal, the fact that our results are in good agreement with their experimental counterparts (particularly in terms of the estimation of *T*_g_ for the two different mixtures, as explained in the next section) gave us confidence in the reliability of the force field. We also note that we have recently validated the usage of the CGenFF force field in the context of a diverse portfolio of molecular glasses.^28^

The GROMACS MD package (version 2022.3, GPU-accelerated, single precision) was used to perform the MD simulations.^29^ We have used a leap-frog integrator to propagate Newton's equations of motion, with a timestep of 2 fs.^30^ A cutoff of 1.2 nm has been used to deal with non-bonded interactions, albeit the forces relative to the van der Waals interactions have been smoothly switched to zero between 1.0 and 1.2 nm. Stochastic velocity and (isotropic) cell rescaling was used to enforce the desired conditions of temperature and pressure, with time constants of 1 and 2 ps, respectively.^31,32^ The LINCS algorithm was used to constrain the length of intra-molecular covalent bonds involving hydrogen atoms, due to the high vibrational frequency of the latter.^33^

We conducted a preliminary geometry optimization (maximum force acting on any atom = 100 kJ mol^−1^ nm^−1^) of the systems, followed by an initial equilibration of 100 ns at 800 K and ambient pressure (NPT ensemble) to ensure the adequate mixing of the components. Then, we quenched the mixtures from 800 to 200 K in 500 ns (NPT ensemble still). Specifically, we followed a “step-like” cooling protocol we have previously validated in ref. 28: for each step (12 steps in total), we linearly decreased the temperature of the mixtures by 50 K in 20 ns, followed by 20 ns of equilibration at that given temperature.

The analysis of the resulting trajectories has been performed *via* in-house Python scripts leveraging the MDAnalysis package.^34^ The hydrogen bonds between the different molecular species have been identified according to the following geometric criteria: (1) the distance between the acceptor and the hydrogen is <3 Å; (2) the donor–hydrogen–acceptor angle is >120°.

## Results and discussion

**Fig. 1:**
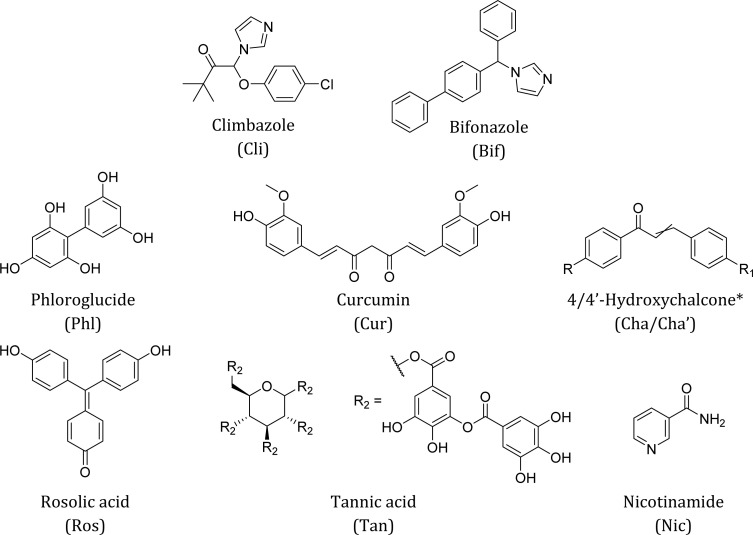
Molecular structure and abbreviations of compounds used to produce binary eutectic molecular liquids and glasses. *For 4-hydroxychalcone, R = H and R_1_ = OH. The reverse is true for 4′-hydroxychalcone.

The overall aim of our work is to show that organic deep eutectic solvents (DES) and their glasses are a candidate platform to replace thermoplastic polymers in certain applications. To find binary mixtures of small organic molecules which form eutectic glasses, pairs of ‘DES-like’ hydrogen bond donor–acceptor pairs were screened. An intentional bias towards hydrophobic molecules was made so the resultant materials would be sparingly soluble in aqueous formulations. Apparent eutectic compositions were determined through heating small-molecule organic mixtures and observing formation of a liquid phase below the melting points of the respective components. Some liquids showed a glass transition near to or above room temperature. In general, from our initial screening two classes of glass were produced – those containing climbazole (Cli) and those containing bifonazole (Bif) (Fig. 1). Accompanying each is a second compound, producing a binary molecular glass close to ambient temperature, and DES at elevated temperatures with unique properties in each case. One hydrophilic system containing nicotinamide (Nic) and tannic acid (Tan) was produced too. A number of these compounds are found in nature and possess favourable toxicological profiles. In our study it was not the objective to find pairs comprised of entirely natural and totally benign compounds. Our emphasis lies on the opportunities these small-molecule eutectic systems can provide as option to replace polymers as material. Given the library of possible binary combinations for DES was estimated between 10^6^–10^8^, there is certainly scope for such pairs to exist.^22,35^

**Fig. 2:**
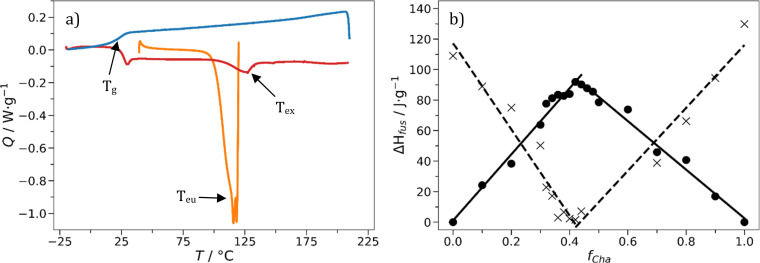
(a) DSC thermogram of off-eutectic Cha/Bif (*f*_Cha_ = 0.34) showing heat flow (*Q*) against temperature (*T*). The eutectic (*T*_eu_, first heating step, 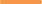 orange line) and excess (*T*_ex_, second heating step, 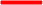 red line) melting peaks are indicated, along with the glass transition (*T*_g_, second cooling step, 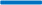 blue line). All other steps are omitted for clarity. (b) Tammann diagram of Cha/Bif showing the eutectic (●, — black line) and excess (×, - - - dashed line) enthalpy of fusion (Δ*H*_fus_) as a function of Cha mole fraction (*f*_Cha_) in a mixture with Bif. Least squares regression is applied to each linear portion of the plot and indicated as solid and dotted lines (see Experimental for details).

Following initial screening, the eutectic mole fraction, *f*_eu_, and glass transition temperature, *T*_g_, of each glass was determined using differential scanning calorimetry (DSC). The eutectic composition is that with the lowest melting point (lower than the melting points of the individual crystalline compounds) and the one that contains no excess crystalline material. The presence of crystalline material would interfere with rheological properties, optical properties through scattering and potentially act as seed nuclei to promote crystallization of the amorphous phase. Two phenomena occur in the first heating step of a typical DSC experiment, as shown in Fig. 2a for the mixture of Cha/Bif. Starting from a blend of the two crystalline starting materials, heating from 25 °C first produces an endothermic peak corresponding to the formation of the liquid eutectic phase at *T*_eu_. Continued heating produces a second, cryoscopically depressed, endothermic peak belonging to the excess crystalline material (*T*_ex_) dissolving into the eutectic liquid. In our example this is Cha (*T*_m_ = 183–185 °C) for mixtures >*f*_eu_, and Bif (*T*_m_ = 149 °C) for compositions <*f*_eu_. The melt and dissolution enthalpies are plotted as a function of composition to produce a characteristic Tammann diagram (Fig. 2b) from which the values for *f*_eu_ are determined by linear data fitting (see Fig. S5–S11†). The main benefit of this approach is it eliminates reliance on peak maxima positions and the excess dissolution peak; the eutectic composition can be determined in the absence of any excess melt data. This is particularly useful as Tan, Phl and Ros (tannic acid, phloroglucide and rosolic acid) all decompose before dissolution; hence they present no excess peak. The eutectic composition of our series of mixtures was found between *f*_eu_ = 0.17–0.42, the two enthalpic maxima/minima from which these mole fractions are derived were generally in good agreement. For example, the *f*_eu_-values for Cha/Bif were 0.42 and 0.43, respectively. These values refer to the mole fraction of the minor component, in this case, Cha.

It is worth noting that obtaining the relevant DSC data outputs for the excess enthalpy values close to the eutectic point is not straightforward. Here the enthalpies resulting from excess material dissolving is small and often indiscernible from noise. Even when peaks are present, there is often ambiguity determining peak positions due to crystal perfection processes. Crystal perfection is the process of the smallest, most imperfect crystals melting below the thermodynamic melting point, subsequently crystallizing, and remelting one or more times as the temperature increases.^36^ This manifests as endothermic melting signals with one or more ‘chunks’ taken out of them. High viscosity liquids are known to kinetically restrict crystal growth, leading to lower melting non-equilibrium structures.^37^ Additionally, away from the eutectic composition excess material can be dissolved into the eutectic phase by raising the temperature above that of the liquidus line in the phase diagram.^38^ Upon cooling, this excess material supersaturates and can crystallize. The timescale in which this crystallization occurs regularly exceeds the timescales of the DSC measurements. This explains why a dissolution peak is not observed after the first heating segment.^39–41^ This supersaturation is the reason stated by Sangoro and co-workers that supercooling DES have been largely avoided in solvent applications.^22^

Values for *T*_g_ are obtained from the second cooling step of the same experiment (Table 1) and for our eutectic systems were found to lie in the range 0–45 °C. It was observed that materials with a *T*_g_ < 20 °C were deformable by hand under applied force, but otherwise largely non-flowing. This indicates high viscosities when the materials were effectively DES, with potentially low kinetic fragility indices (*m*). This is supported by the relatively wide glass transition regions, for example 13 °C for Cha/Bif, often indicative of low fragility materials.^42,43^ This property contrasts with that of polymers, whose fragility index is typically high. This feature and its implications will be discussed later in the manuscript.

**Table 1:** Summary of eutectic glass properties

Sample	*f*_eu_ a	*T*_g,e_ b/°C	*T*_g,m_ b/°C	*T*_g,o_ b/°C	*T*_rg_ c/°C	*m* d/—	*t*_st_/mos^e^
Cur/Cli	0.22	−2.3	8.4	19.2	0.8	8.7	>7
Phl/Cli	0.17	−8.1	−1.2	5.4	0.7	8.5	0.75
Ros/Cli	0.17	7.4	12.9	18.3	0.8	4.7	0.5
Cha/Bif	0.42	16.4	22.9	29.5	0.7	17.4	>14
Cha′/Bif	0.42	16.5	22.9	29.2	0.8	17.0	>4
Cur/Bif	0.35	33.9	42.2	50.8	0.8	16.5	>4
Tan/Nic	0.31	−9.6	−1.4	6.8	0.7	—f	>4

a Mole fraction of the first compound wrt. The second compound in the eutectic phase (*f*_eu_), as written.

b Subscript refers to the onset, midpoint, and end of the glass transition.

c Reduced glass transition temperature (*T*_rg_) calculated from *T*_g,o_/*T*_eu_.

d Kinetic fragility index (*m*) as defined in eqn (3).

e Stability (*t*_st_) refers to the time elapsed in months (mos) before crystallization peaks appeared in each XRD pattern. Note each material was measured discontinuously for different lengths of time, and most did not fully crystallize within the experimental timescale.

f Difficult to obtain due to Nic sublimation.

**Fig. 3:**
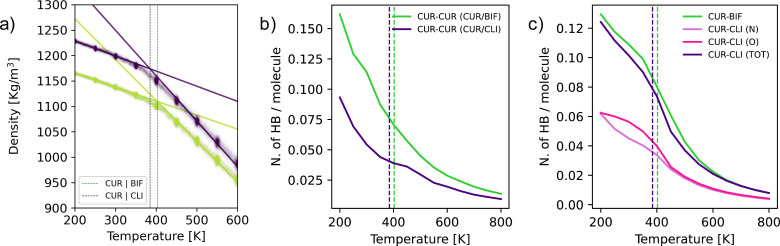
(a) Computational estimate of the *T*_g_ of the Cur/Bif (403 ± 6 K, green vertical line) and Cur/Cli (385 ± 5, purple vertical line) mixtures. (b) Number of curcumin–curcumin hydrogen bonds (per molecule) for the Cur/Bif and Cur/Cli mixture as a function of temperature. (c) Number of curcumin–bifonazole/climbazole hydrogen bonds (per molecule) for the Cur/Bif and Cur/Cli mixture as a function of temperature.

To investigate the origins of the significant spread in terms of *T*_g_ observed for the different mixtures (see Table 1), we have performed MD simulations, focussing on the Cur/Bif and Cur/Cli mixtures. The computational details are discussed in the Experimental section. In short, we have quenched both mixtures from the melt (800 K) to 200 K. As illustrated in Fig. 3a, the intersection between the linear fits of the density *versus* temperature data for the liquid (500–800 K) and the glass (200–300 K) phase provides an estimate of the *T*_g_ of the two mixtures. We obtain *T*_g_ = 403 ± 6 K and 385 ± 5 K for Cur/Bif and Cur/Cli, respectively, to be compared with the experimental midpoints of 315 K and 281 K. The fact that our computational estimates substantially overestimate the *T*_g_ is expected, as the computational cooling rates (∼1 K ns^−1^ in this case) are orders of magnitude higher than their experimental counterparts (∼2 × 10^−10^ K ns^−1^ in this case). Nevertheless, our simulations qualitatively capture the experimental trend in terms of the *T*_g_ of these two mixtures, despite the sub-optimal parametrization of the force field we have used (see the Experimental section). This result is in line with our recent experience with computing the *T*_g_ for a large number of molecular glasses and gives us confidence in the reliability of our computational results.^28^ Interestingly, the Cur/Bif mixture appears to be substantially less dense than Cur/Cli, with this difference being particularly evident below *T*_g_. Moreover, the thermal expansion (or, equivalently, the density evolution) of the two systems is very similar below *T*_g_, with substantial differences in the behaviour of the supercooled liquid phase instead (see the rather different slope of the two sets of results above *T*_g_).

As a first step to understand the structural differences between the Cur/Bif and Cur/Cli mixtures, we have computed the pair correlation functions (PCFs) for these systems (utilising the centres of mass for each molecule) as a function of temperature. The results are reported in the Fig. S4.† Interestingly, we find that most of the structural changes within the mixtures as we cooled down the liquid into the glass concern the curcumin molecules – even though their molar fraction within the mixtures is quite low (0.35 or 0.22) compared to either bifonazole or climbazole. Whilst we do observe some changes in the Cur–Bif and Cur–Cli PCFs as well, it is the Cur–Cur PCF that it is indicative of greater structuring for Cur/Bif compared to Cur/Cli.

Given that all the molecular species involved in these mixtures are capable to form hydrogen bonds (HBs) with each other, we asked ourselves whether the structural differences we observed *via* the PCFs might originate to some extent from different hydrogen bond networks within the two mixtures. As it can be seen from Fig. 3b, where we reported the number of Cur–Cur HBs (per molecule) as a function of temperature for the two mixtures, it appears that Cur molecules develop a significantly higher number of HBs in the case of Cur/Bif compared to Cur/Cli. Note that, whilst the mole fraction of Cur is higher for Cur/Bif (0.35) than it is for Cur/Cli (0.22), the number of Cur–Cur HBs is very similar for both mixtures in the liquid phase at high temperature (*e.g.*, 800 K). The differences in the HB network seems to be developing as we approach *T*_g_ – and thus persist within the glass as well. Interestingly, the number of Cur–Bif and Cur–Cli HBs, reported in Fig. 3c (again per molecule, and again as a function of temperature), is overall very similar for the two mixtures. Note that Cli molecules present two different acceptors, N and O – but the total number of Cur–Cli HBs is very similar to that of Cur–Bif across the whole temperature range. This result is consistent with the fact that the structuring of the Cur–Bif and Cur–Cli PCFs (see Fig. S4†) is rather similar, whilst the main difference lies in the structuring of the Cur–Cur PCFs, which we argue originates in part from the different HB network within the two mixtures.

Whilst we are in no position to claim that differences in the HB network are at the heart of every single mixture investigated in this work, we argue that acting on the number of HB acceptors and donors within the molecular species involved in the formation of these binary mixtures might present an interesting opportunity to tailor their *T*_g_ and potentially their physical stability as well (albeit the two are not simply proportional to each other, as discussed later).

Once an optimal and thus eutectic recipe is known, the crystalline starting materials can be converted to glassy products reproducibly in practically quantitative yield simply by heating and mixing for <1 hour. The mixing step is important as initially direct contact between the surfaces of both crystals is essential. Our systems were small-scale, typically up to 5.0 g. Scale-up routes can be ball-milling or extrusion, the latter we briefly piloted using a mini-extruder (Fig. S12†). Given the right starting materials, the resultant glass has the potential to be environmentally benign, biodegradable, and recyclable.

Eutectic glasses containing curcumin (Cur/Cli and Cur/Bif) always contained a small quantity of residual orange powder. ‘Curcumin’ is generally sold as a mixture of three structurally similar curcuminoids – 75–85% curcumin, 10–20% desmethoxycurcumin and <5% bisdesmethoxycurcumin. Plausibly the residual orange powder implies the formation of a more complex multi-component eutectic system that is off its eutectic composition.

The chemical nature of the molecules in this study inspired us to validate the eutectic composition using visible light absorbance spectroscopy. This was performed for the Cha/Bif system. Mixtures at *f*_eu_ = 30, 40 and 50 were heated at *T*_eu_, that is 116 °C, as determined by DSC. A portion of the liquid eutectic phase free from excess solids was isolated in a similar manner as reported by Margerum and co-workers.^44^ The liquid samples were dissolved in ethanol and their UV-vis spectra were recorded between 200–800 nm. Concentrations of the individual components were obtained through calibration using stock solutions of either Cha or Bif, and their mixtures (Table S1†).

A partial least squares regression (PLSR) supervised machine learning model was used for data processing and analysis. The main benefits of using PLSR are that absorbance data at all wavelengths are accounted for during calibration, rather than one fixed wavelength (often *λ*_max_), as done traditionally. Deconvolution of overlapping absorption peaks is also trivialized. This is particularly useful as Cha and Bif both have absorption bands near 256 nm. The model is trained on calibration data of Cha/Bif solutions at various known concentrations of pure Cha or Bif, and their mixtures. In our case 50 independently prepared solutions were used as calibration training set. The predictive accuracy of the PLSR model was evaluated using *k*-fold cross-validation (*k* = 5). Here the dataset is randomly split into 5 subsets. The model is trained using 4 subsets and the performance estimated using the remaining subset. This process is repeated 5 times and the performance taken as an average *R*^2^ over all folds. This gave *R*^2^ = 0.98 (*σ* = 0.01), indicating accurate predictive performance. The optimal number of latent variables was determined as 4, indicated by a minimum in the root-mean-square error of cross-validation (RMSECV), visualized in Fig. S2.† A typical ‘elbow-shaped’ relation is exhibited. *K*-Fold cross-validation is crucial for small to medium sized datasets, as is the case here (50 samples). Splitting a small dataset can introduce significant bias as the split chosen is not necessarily representative.^24^ Different splits would therefore yield different performance scores. *K*-Fold cross-validation minimises this bias.

Feeding the spectral data acquired from our unknown eutectic samples into the trained model outputs predicted concentrations of Cha and Bif in solution. This was applied to the isolated eutectic phase, yielding an average predicted composition of 44 mol% Cha, in good agreement with the value of 42 mol% as obtained by DSC.

Long-term stability of eutectic molecular glasses towards crystallization was probed using powder X-ray diffraction (XRD). Stability against time is a make-or-break property for product development and often overlooked. If you make a glass with the desired properties, except that it is unstable and reverts to crystallinity on prolonged timescales, it is largely useless. Depending on the application, a product may require a shelf-life of months, or even years.^45^ It is therefore crucial its components remain stable over this duration.

**Fig. 4:**
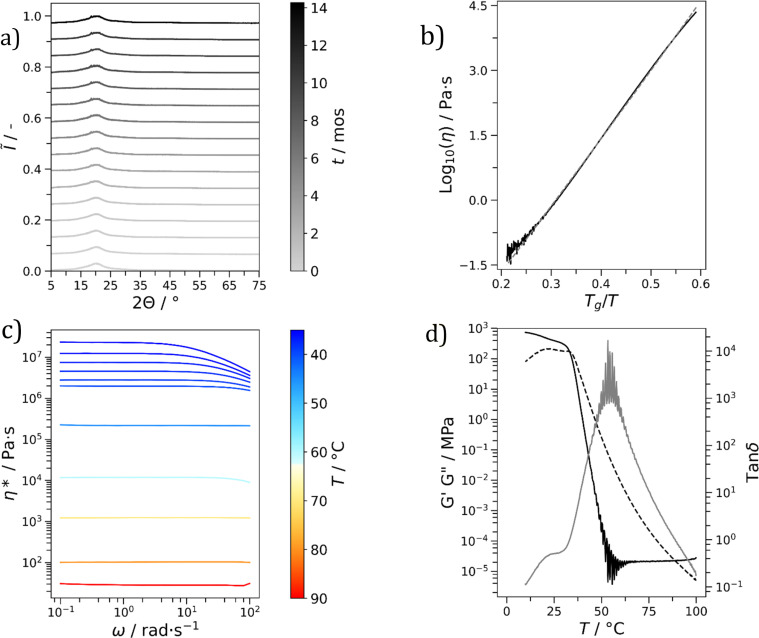
Cha/Bif (a) XRD patterns over 14 months (mos) demonstrating good stability of the broad amorphous phase against crystallization at room temperature. Intensities are Min–Max normalized (*Ĩ*) so values lie between 0 and 1 and offset by *Ĩ* = 0.066. (b) Angell plot showing the relationship between log_10_(viscosity) (*η*) and *T*_g_/*T*. A VFT fit is overlayed (---, dashed line). (c) Frequency sweeps demonstrating shear-thinning reduction in complex viscosity (η*) at low temperature (*T*) and high angular frequency (*ω*). (d) Relationship of tan *δ* (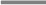, grey line) and the storage (*G*′, —, black line) and loss (*G*′′, - - -, dashed line) moduli against *T* using oscillatory rheology.

The stability of each glass (see Table 1) was monitored over a period of months (Fig. S13–S18†). It is important to emphasize that this period was variable, with some materials being monitored for longer than others. A spread of stabilities was found, with Cha/Bif, Cur/Cli, Cha′/Bif and Cur/Bif remaining stable for at least the duration of the experiment: 14, 7, 4 and 4 months, respectively. Here the XRD patterns displayed broad, characteristically amorphous peaks with no changes in shape and position throughout the monitoring period (Fig. 4a, S12 & S15–16,† respectively). The XRD patterns of the eutectic binary glass Cha/Bif were recorded at different storage times, up to 14 months (room temperature, not thermostated). Two broad peaks are present, the most prominent at 20° and the other at 44°. Small peaks at 18.5 and 26.6° appeared after ∼1 month. These could be a result of either noise or crystallisation. The latter is more likely considering their consistent presence from 3 months onward. The XRD pattern of pure Cha has peaks at 18.6 and 26.6°, suggesting Cha is crystallizing (see Fig. S19–S27†). The peaks do not grow to any significant extent over time, showing crystallization here is slow. An alternative explanation could be the initial composition was slightly off-eutectic. Here crystallization would drive the composition towards the eutectic and cease once reached.

The peak shape and position are caused by localized ordering.^46,47^ We observed in all of our binary mixtures that cooling from a liquid state towards *T*_g_ leads to volumetric contraction. In combination with a reduction in molecular motion, this allows molecules to pack more closely, simultaneously phasing-in short-range repulsive forces. This gives rise to a mosaic of locally ordered regions, each contributing a distinct diffraction profile. The experimentally measured diffraction pattern represents an average of this local order.

The prolonged stability of the small molecule eutectic binary glasses is in line with the calculated reduced glass transition temperature (*T*_rg_) of each material (Table 1). The *T*_rg_ values all satisfy Kauzmann's 2/3 rule (eqn (1)‡‡Eqn (1). Kauzmann's equation for the reduced glass transition temperature (*T*_rg_). *T*_g,o_ and *T*_m_ refer to the glass transition onset temperature and the melting temperature (in this case, of the eutectic; *T*_m_ = *T*_eu_), respectively.) where values above this threshold indicate good resistance of a supercooled liquid towards crystallization and high glass-forming ability (GFA).^48^ Materials of high GFA have been shown to remain stable even when seeded with crystallites.^49^1
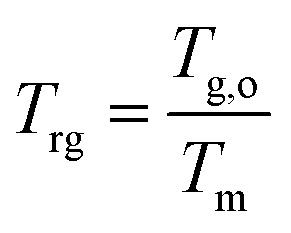


As can be seen from Table 1, two of the systems investigated, that is Phl/Cli and Ros/Cli, proved significantly less stable. After around two weeks and three weeks, respectively, the samples began to exhibit significant crystallization (Fig. S14 and S15†). This is marked by the evolution of broad, amorphous peaks to sharp, crystalline peaks. Ros/Cli fully crystallized within 2–3 weeks, while Phl/Cli retained minor amorphous character even up to 7 months. The most intense crystalline peaks were found at 16.3, 20.5 and 25.7 for both samples. Unsurprisingly, the peaks match those found in the XRD pattern of pure Cli (16.2, 20.4 and 25.6°, Fig. S20†). This makes sense, considering Cli constitutes 83% of both binary mixtures by mole fraction.

All eutectic molecular glasses under study showed dependence of viscosity (*η*) on temperature (*T*) (Fig. S28†). Note that viscosities were determined using a parallel plate geometry (8.0 mm top, 50 mm bottom) at a velocity of 1.0 rad s^−1^. The dependence of viscosity on temperature for Cha/Bif is presented in Fig. 4b as an Angell plot.^49^ Our experimental data could be fitted with the Vogel^50^–Fulcher^51^–Tammann^52^ (VFT) equation, with *r*^2^ > 0.999 in all cases (eqn (2)§§Eqn (2). Vogel–Fulcher–Tammann relation.). Here *η*_0_, *B* and *T*_VF_ are empirical fitting parameters (Table S3†).2
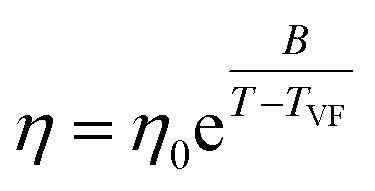


The VFT equation is commonly used to describe the viscosity of glass-forming liquids. Here it enables extrapolation of the rheology data to lower temperatures, those which are inaccessible due to the difficulty shearing such viscous media. Slight deviation from the VFT relation was observed at lower temperatures, which could potentially be explained by the onset of shear thinning. This phenomenon is known to occur in small molecule hydrogen-bonded systems. For example, simulations of ethanol have shown that shear thinning occurs at high shear rate (*γ* = 0.0025 ps^−1^, 295 K) due to disruption of hydrogen bonding.^53^ Values for the viscosity at *T*_g_ (mid-point values from DSC) can be obtained from the extrapolated data and used to calculate the kinetic fragility index, *m*, defined in eqn (3).¶¶Eqn (3). Kinetic fragility index (*m*). Put simply, this is the gradient of a log_10_(*η*) *vs. T*_g_/*T* plot where *T* = *T*_g,e_.3
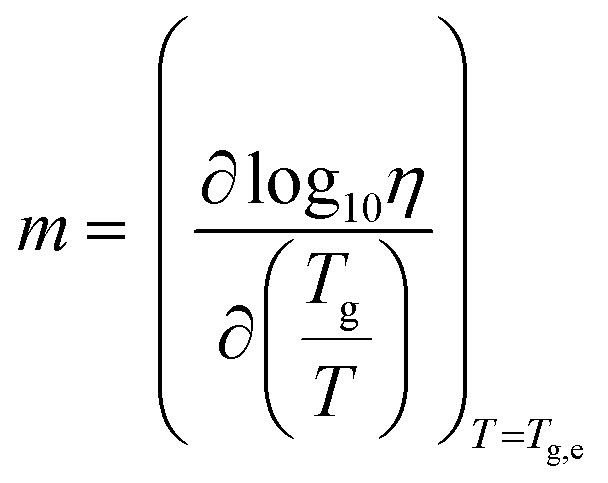


Low fragility indices were found for all materials, with values lying in the range 4.7–17.4 (Table 1). This agrees with the widths of the glass transition regions found with DSC which were suggestive of low fragility.^42,43^ In the physical sense, low fragility means viscosity or structural relaxation time changes relatively slowly with temperature. With Cha/Bif as an example, *T*_g,m_ is 22.9 °C, yet viscosity at 60 °C is still 14 000 Pa s. Only at temperatures >115 °C does viscosity approach the sensitivity of the instrument and geometry (∼5 Pa s), whereafter the data becomes noisy. These results are comparable to SiO_2_, the primary component of window glass, with *m* = 20.^49^ Wang and co-workers showed fragility is often depressed in binary and ternary glass forming eutectic mixtures (although the minima is offset from the eutectic composition).^54^ Both this and the nature of the molecular interactions are likely responsible for the low fragilities observed.^55^

**Fig. 5:**
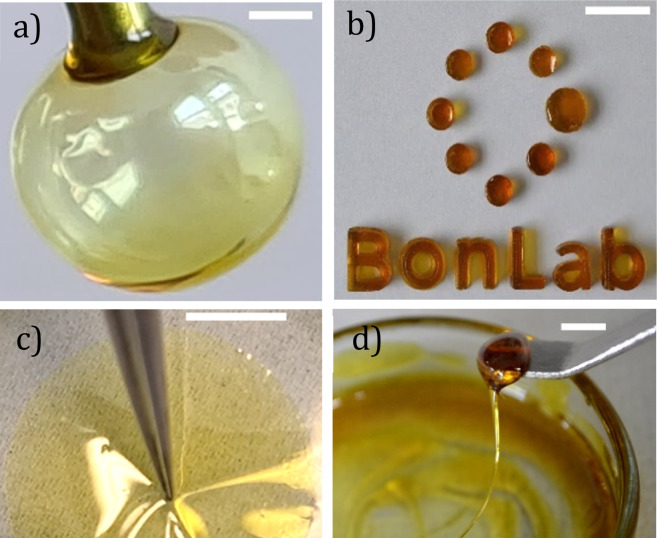
(a) Bubble of Cha/Bif eutectic glass. (b) Molding of Cha/Bif into complex shapes. (c) Formation of a Cha/Bif glass film by deposition from EtOAc (33.9 mg g^−1^) partitioned above EtOAc-saturated water. Flexibility is demonstrated by physical deformation. (d) Pulling of a Cha/Bif glass fiber. Scale bars = 1 mm (a–c) and 5 mm (d).

The main benefit of low-fragility materials is their advanced processability in the form of glass blowing. This is less accessible to polymers, whose fragilities are commonly high (*e.g. m* ∼ 160 for polystyrene with *M*_n_ = 10^5^ g mol^−1^).^56^Fig. 5a demonstrates this, showing a bubble blown from a flat-headed syringe needle. The bubble is comprised of a shell of glassy Cha/Bif and a core of encapsulated air. The ability to glass-blow these materials means they can easily be formed into complex shapes. Like a polymer melt, it is also possible to mould these glasses, as shown in Fig. 5b. Interestingly, thin films can be produced with ease by deposition of glass from a volatile phase such as ethyl acetate (Fig. 5c and ESI Video†). Flexible fibres can also be drawn, as shown in Fig. 5d. The flexible processability of eutectic molecular glasses is a useful trait which would enable them to be sculpted to meet the needs of many applications.

The rheological properties of each material in the molten state were probed to identify any non-Newtonian flow behaviour. One desirable example would be shear-thinning. This is a phenomenon characterized by a reduction in viscosity as a function of increasing shear rate. A reduction in viscosity at high shear rate would be beneficial for processability. To probe for this behaviour, frequency sweeps of each material were performed within a predetermined linear viscoelastic region (10^−2^–10^2^% strain, LVER, Fig. S29†). The frequency sweeps for Cha/Bif are shown in Fig. 4c. Newtonian behaviour was observed for all materials at low oscillation frequencies, independent of temperature (Fig. S30–S35†). At high frequency and low temperature, non-Newtonian, shear-thinning behaviour, was observed for Cha/Bif (2.5 rad s^−1^, 35 °C), Cur/Cli (1.6 rad s^−1^, 30 °C), Cur/Bif (2.0 rad s^−1^, 55 °C) and Phl/Cli (7.9 rad s^−1^, 30 °C). Similar behaviour has been reported previously in triazine-based molecular glasses by Lebel and co-workers.^57^ They ascribed this behaviour to shear-induced disruption of hydrogen bonding in supramolecular aggregates. It is possible a similar shear-thinning mechanism exists in the present study. Shear forces act to disrupt sample microstructure, while low temperature slows both molecular mobility and structural relaxation. Both conditions therefore promote shear-thinning under this hypothesis.

**Fig. 6:**
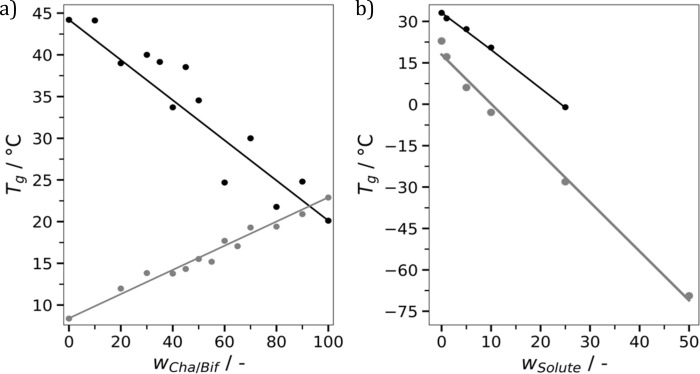
(a) Dependence of *T*_g_ on the weight fraction of Cha/Bif (*w*_Cha/Bif_) compared with Cur/Bif (black) and Cur/Cli (grey). Solid lines correspond to the Gordon–Taylor fit (*k* = 1 in all cases). (b) Effect of texanol (black) and lavandin oil (grey) mass fraction (*w*_solute_) on the *T*_g_ of Cha/Bif. Texanol data was determined using rheology and fit with the Gordon–Taylor equation (*k* = 0.84). Lavandin oil data was acquired using DSC and fit by linear regression.

One of the major advantages of polymer materials is their tunability. Adjusting factors such as molecular weight, monomer composition and degree of crosslinking enables tailored properties. This makes polymers highly versatile and adaptable compared to many other materials. This made us wonder, is it possible to tune the properties of molecular glasses in a similar fashion? For example, if you mix two molecular glasses together, do you get intermediate properties? This was investigated first for mixtures of Cha/Bif and Cur/Bif using DSC (Fig. 6a). A linear relationship (*r*^2^ = 0.89) between composition and *T*_g_ was found. Due to the shared Bif component, this system could potentially be considered a ternary eutectic. We therefore confirmed this behaviour for mixtures of Cha/Bif and Cur/Cli, again finding a linear relationship (*r*^2^ = 0.97).

Plasticization is another method commonly employed to tune the properties of polymers. Plasticization lowers the *T*_g_ which in turn improves processability. We envisioned this would be possible with molecular glasses, not least because binary eutectics like DES are routinely used for this purpose. To investigate this, mixtures of Cha/Bif with varied quantities of texanol were probed using oscillatory temperature sweeps (see Fig. S36† for all glasses without texanol). Texanol is a benign small molecule ester alcohol commonly used as a plasticizer in the paint and coatings industry. A cross-over of the storage (*G*′) and loss (*G′′*) moduli indicates a phase transition – in this case, the *T*_g_. A linear relationship (*r*^2^ = 0.99) between texanol weight fraction (*w*_Tex_) and *T*_g_ was found, as shown in Fig. 6b. The Gordon–Taylor fit (eqn (4),||||Eqn (4). Gordon–Taylor equation describing the *T*_g_ of miscible binary mixtures.*k* = 0.84) is overlayed and describes the relationship well. This is an equation often used for predicting the *T*_g_ of miscible binary mixtures (*T*_g_,_mix_), where *T*_g1_, *T*_g2_ and *w*_1_, *w*_2_ are the *T*_g_'s and weight fractions of components 1 and 2, respectively.^58^ This shows that *T*_g_ and associated properties such as viscosity can be tuned easily and predictably by addition of a third component.4
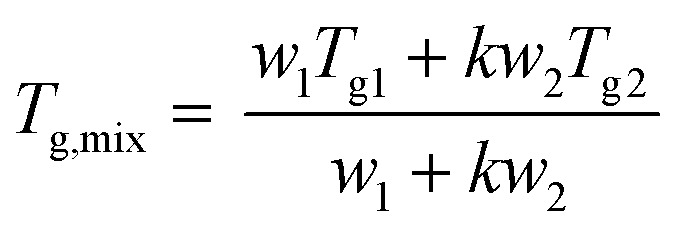


**Fig. 7:**
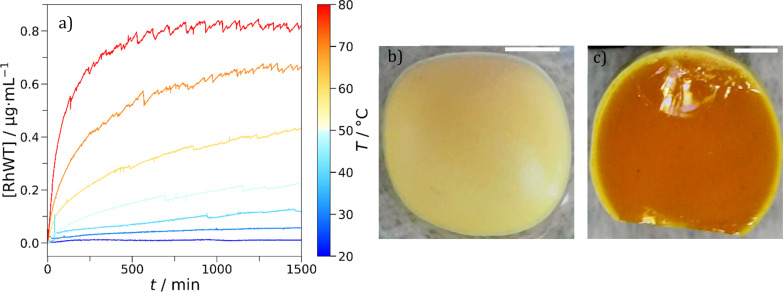
(a) Release of rhodamine WT (RhWT) from Cha/Bif glass, measured as a function of temperature (*T*) and time (*t*) using UV-vis absorbance spectroscopy (556 nm). (b) A different Cha/Bif particle after being submerged in water for a week. (c) Cross-section of the opaque Cha/Bif particle. The opaque crystalline shell is approximately 150 μm thick. The inner core remains as it were at *t*_0_. Scale bars: 1 mm.

Colloidal delivery systems are one application where we hypothesized eutectic molecular glasses would excel. We need to remember that the beneficial agent or drug can influence the properties of the system. For example, the addition of lavandin oil to Cha/Bif lowered the glass transition temperature (Fig. 6b, grey data.) We studied the storage and release characteristics of the Cha/Bif system by dissolving a UV-active dye, rhodamine WT (RhWT) in molten Cha/Bif. The resultant dye-loaded liquid was cast as a film (100 mg) at the base of a cuvette (10 × 10 mm), cooled to room temperature and water added thereafter. The diffusion of RhWT from Cha/Bif glass into water was measured as a function of temperature and time using UV-vis absorption spectroscopy, as shown in Fig. 7a. Between 20–40 °C, very little release is observed because *T* ≈ *T*_g,e_ (29.5 °C). This is expected because the low fragility, high viscosity nature of these eutectic molecular glasses means molecular diffusion is severely restricted close to, or below, *T*_g_. This inverse proportionality is evident in the Stokes–Einstein–Sutherland equation (eqn (5)**Eqn (5). Stokes–Einstein–Sutherland equation describing the diffusion of a sphere through a liquid. *D*, *k*_B_, *T*, *η* and *r* are the diffusion coefficient, Boltzmann constant, temperature, viscosity, and radius respectively.):5
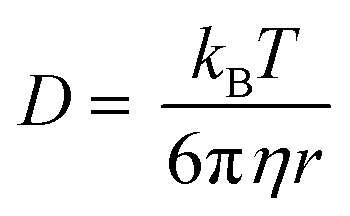


The dependence of viscosity on temperature enables tunable encapsulate release. Increasing the temperature of the system correspondingly increases the rate of dye release into the water phase. This is aided by the low fragility of these materials, which provides a larger window in which viscosity and therefore diffusion can be tuned.

What came as an initial surprise was that compared to the theoretical RhWT content in the film (39.5 μg) relatively little was released, even at 80 °C (11.4%). This is far below its solubility limit of 0.4 g per 2 g of water. Moreover, the release profiles at higher temperatures seem to indicate that release is gradually arrested. This suggests that a different phenomenon apart from stagnant Fickian diffusion plays a role. The former would only make sense if absorbance continued to rise slowly over time in the release experiments. Instead, we find it approaches a constant value on the timescale of hours. We therefore had a closer look at the interface and found that it had become opaque. This is explained and consistent with a partial eutectic matrix dissolution influencing the release mechanism. Despite their hydrophobicity, both Cha and Bif have a small, but finite water solubility (240.3 and 2.45 mg L^−1^ respectively, at room temperature). Cha is around 100× more water soluble, so should dissolve in preference to Bif. What happens is that its dissolution triggers crystallization of Bif at the interface thereby forming a barrier layer. This was experimentally observed in that following release experiments a thin, white deposit (Fig. 7b and c) is present on the surface. In terms of crystal color appearance, Cha is yellow and Bif is white, so this supports the idea of Cha dissolving preferentially and saturating the solution.

## Conclusions

We showed that hydrophobic eutectic molecular glasses and liquids are an interesting technology platform to potentially replace thermoplastics polymers in certain applications. A series of highly viscous eutectic molecular liquids and glasses were produced. Tendency of the amorphous materials towards crystallization was found to be low in most cases. For example, Cha/Bif was found to be stable in excess of 14 months. This agrees with the stabilities predicted from the Kauzmann 2/3 rule and is beneficial for product shelf-life stability. The shear-thinning nature of the eutectic mixtures is thought to be caused by shear-induced disruption of intermolecular interactions. Further investigation would be required to determine the exact cause. Parallels between eutectic molecular glasses and polymers can be drawn in terms of property tuning. In a blend of two different glasses, the *T*_g_ of the mixture can be tuned by varying the weight fraction. In effect, this enables the viscosity of the mixture to be modulated which in turn broadens the pool of potential applications for these materials. Molecular dynamics (MD) simulations offered insight into the potential origins of the wide range of *T*_g_ values we observed for different mixtures, with emphasis on the role of hydrogen bonding. We argue that MD simulations might be used in future work to rapidly screen a wide array of different mixtures to identify promising candidates with respect to both *T*_g_ and physical stability. We highlighted delivery systems as one potential application where polymers could be replaced. We view our study as a prototype to spur future work which focuses on low-cost, benign eutectic mixtures which can be formulated across a range of applications to offer an alternative to plastic.

## Data availability

Experimental data is available from the ESI,† see DOI: https://doi.org/10.1039/d4sc02574a. Computational data is available on GitHub with the link: https://github.com/BonLab/chemsci2024.

## Author contributions

Conceptualization (JLR, SAFB), data curation (JLR, GCS, SAFB), formal analysis (JLR, GCS, SAFB), funding acquisition (SAFB), investigation (JLR), methodology (JLR, GCS, SAFB), project administration (JLR, SAFB), supervision (SAFB), validation (JLR, GCS, SAFB), visualization (JLR), writing – original draft (JLR, GCS, SAFB) writing – review & editing (JLR, GCS, SAFB).

## Conflicts of interest

There are no conflicts to declare.

## Supplementary Material

SC-OLF-D4SC02574A-s001

SC-OLF-D4SC02574A-s002

SC-OLF-D4SC02574A-s003
