# Supplementary material for: Small molecule organic eutectics as candidates to replace plastics
Source: Chem Sci. 2024 Aug 12;15(35):14458–70. doi: 10.1039/d4sc02574a (PMC11331394; doi:10.1039/d4sc02574a)
Supplement: SC-015-D4SC02574A-s002 [file SC-015-D4SC02574A-s002.pdf]

Supplementary Information (SI) for Chemical Science.
This journal is © The Royal Society of Chemistry 2024

ESI for: Small molecule organic eutectics as candidates to replace plastics.

Josh L. Ryan, Gabriele C. Sosso*, Stefan A. F. Bon*

Department of Chemistry, University of Warwick, Coventry, CV4 7AL, United Kingdom.
S.Bon@warwick.ac.uk G.Sosso@warwick.ac.uk web: https://bonlab.info.

Table of Contents

Figures

Figure S1. Differential Scanning Calorimetry Time-Temperature Plots 1
Figure S2. Partial Least Squares Regression RMSECV vs number of LVs 12
Figure S3. Rhodamine WT Calibration Plots 3
Figure S4. Pair correlation functions of the Cur/Bif and Cur/Cli mixtures 4
Figure S5-11. DSC and Tammann Diagrams 5-11
Figure S12. Mini-extrusion Testing 12
Figure S13-18. XRD Stability vs Time 13-18
Figures S19-27. XRD of Pure Glass Constituents 19-27
Figure S28. Angell Plots 28
Figure S29. Amplitude Sweeps 29
Figure S30-35. Oscillatory Frequency Sweeps 30-35
Figure S36. Oscillatory Temperature Sweeps 36

Tables

Table S1. Molecular Glass Eutectic Temperatures 38
Table S2. Viscosity-temperature Parameters 38
Table S3. Amplitude Sweep Parameters 38
Table S4. Frequency Sweep Parameters 39
Table S5. Oscillatory Temperature Sweep Parameters 39
Table S6. Partial Least Squares Regression Calibration Solutions 37

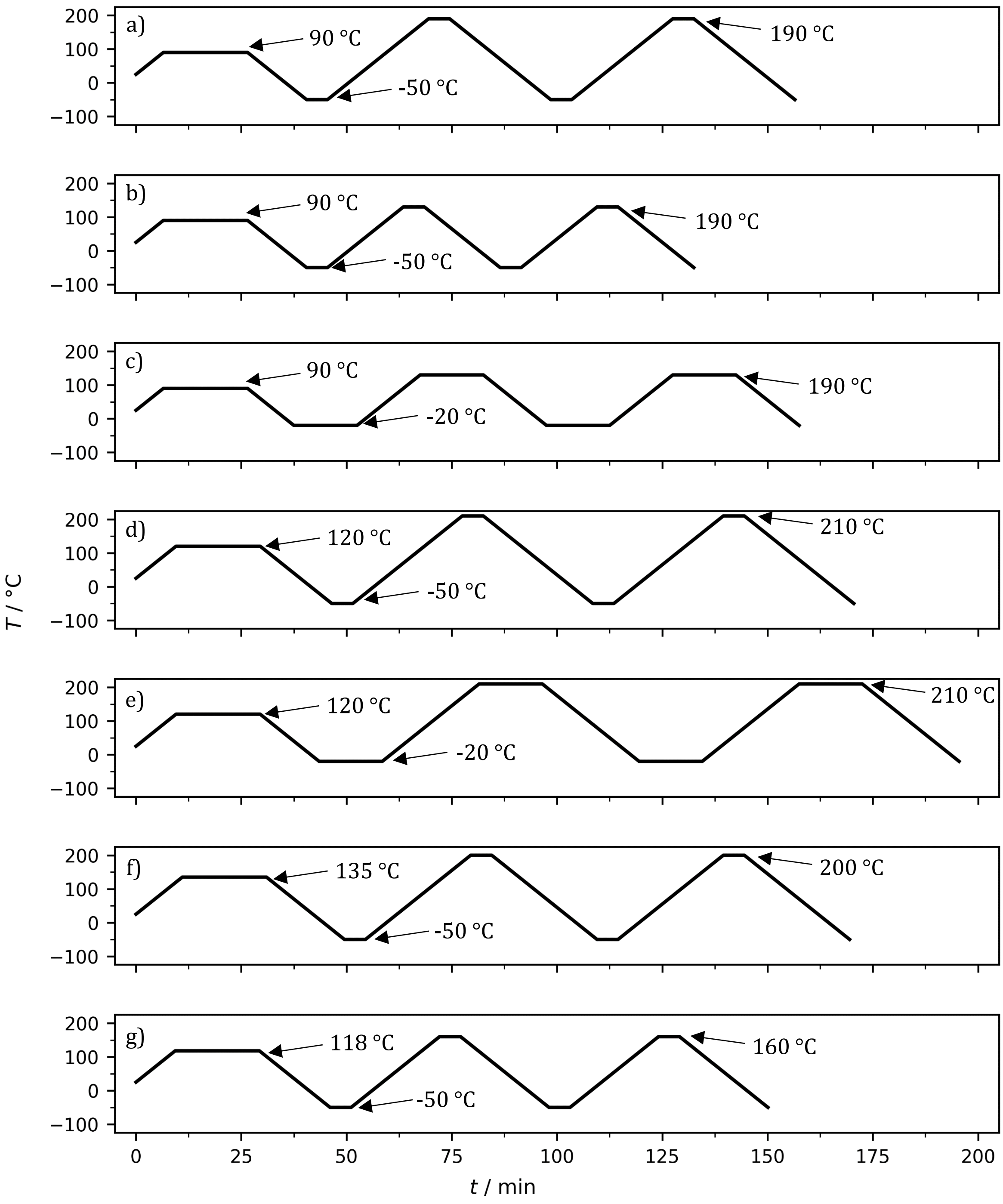

Figure S1. Differential scanning calorimetry time-temperature (t-T) plots for a) Cur/Cli, b) Phl/Cli, c) Ros/Cli, d) Cha/Bif, e) Cha'/Bif, f) Cur/Bif and g) Tan/Nic. The starting temperature (25 °C), ramp rate (10 °C·min^{-1}) and sample size (10 mg) were constant for all samples. All measurements were performed using hermetically sealed aluminum pans. The instrument was calibrated with indium and operated with a nitrogen flow rate of 50 mL·min^{-1}.

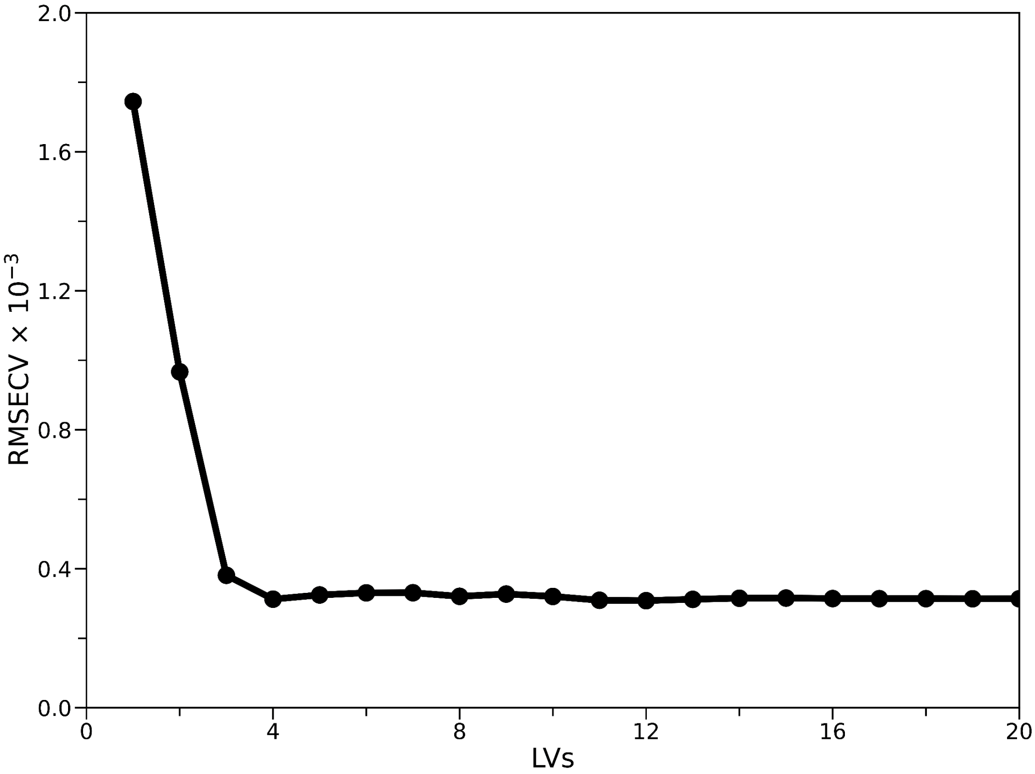

Figure S2. RMSECV vs number of latent variables (LVs). The minimum indicates the optimal number of LVs which should be used to train the PLSR model.

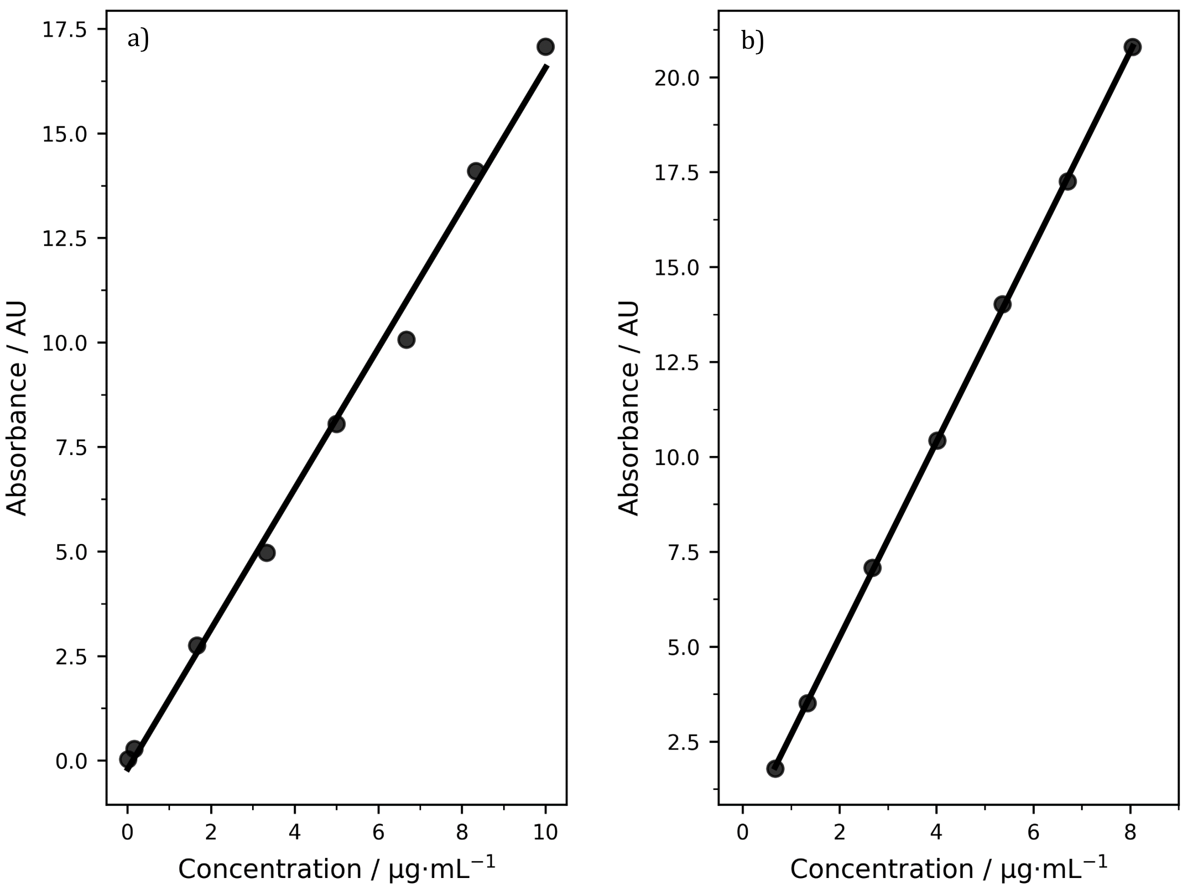

Figure S3. Rhodamine WT (RhWT) in ethanol (a) and water (b) calibration plots of peak area vs. concentration (r^2 = 0.995 and 0.999, respectively).

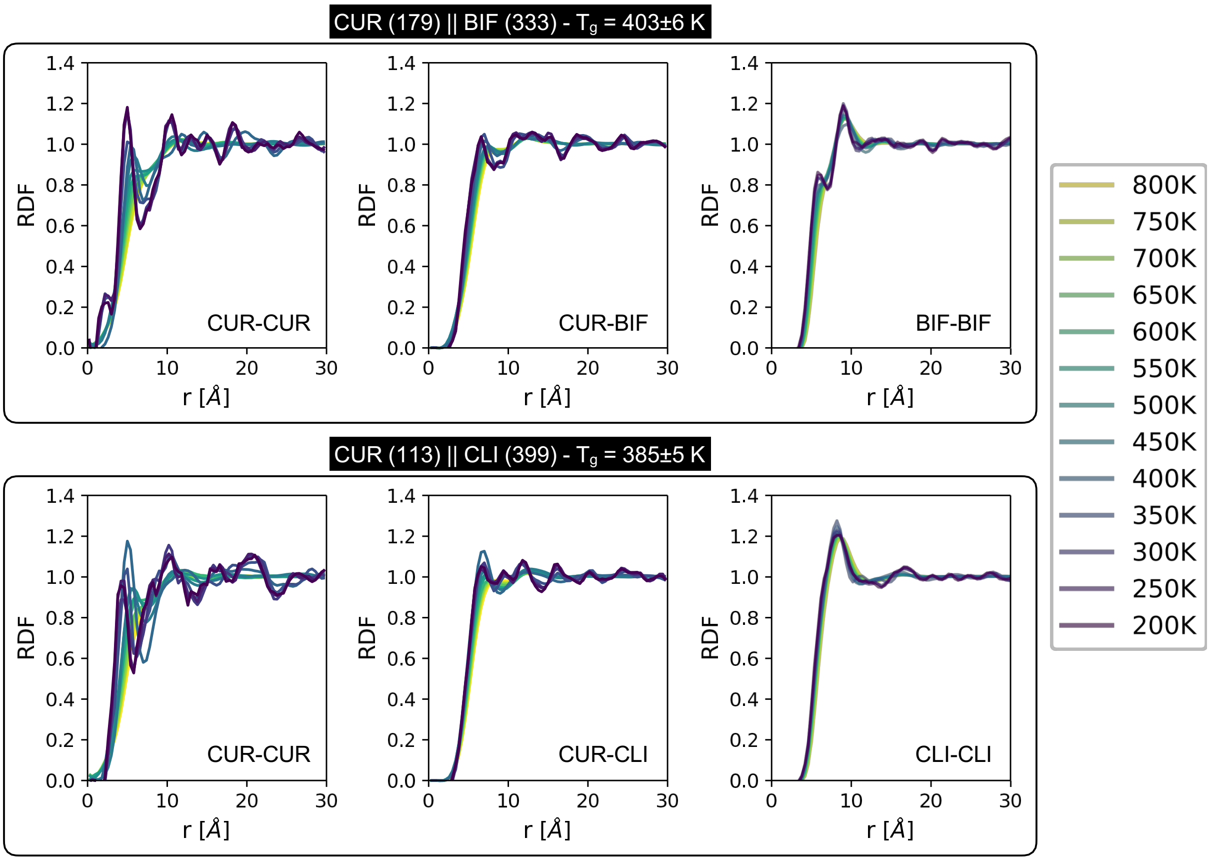

Figure S4. Radial distribution functions (RDF) for the Cur/Bif and Cur/Cli mixtures (see main text) as a function of temperature. Note that for each molecule we have considered its center of mass for the purposes of computing the RDF.

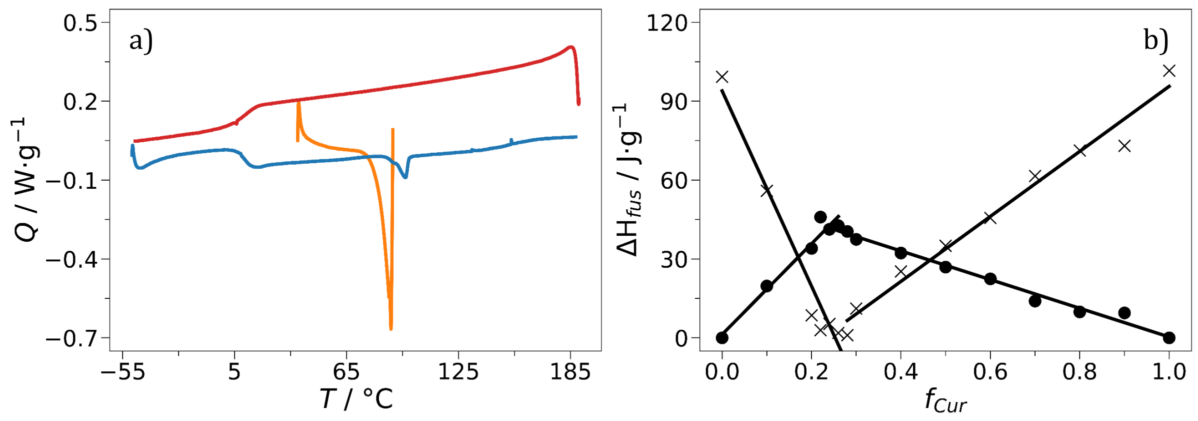

Figure S5. a) DSC thermogram of eutectic Cur/Cli (f_{Cur}= 0.22) showing heat flow (Q) against temperature (T). The eutectic (T_{eu}, first heating step, —) and excess (T_{ex}, second heating step, —) melting peaks are indicated, along with the glass transition (T_g, second cooling step, —). All other steps are omitted for clarity. b) Partial Tammann diagram of Cur/Cli showing the eutectic (●, —) and excess (**×**, **---**) enthalpy of fusion (ΔH_{fus}) as a function of Cur mole fraction (f_{Cur}) in a mixture with Cli. Least squares regression is applied to each linear portion of the plot and indicated as solid and dotted lines.

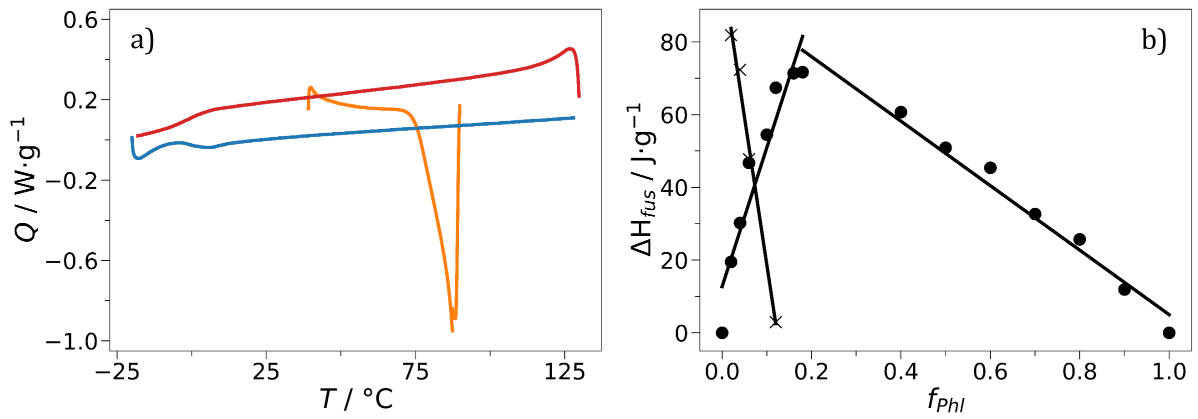

Figure S6. a) DSC thermogram of eutectic Phl/Cli (f_{Phl}= 0.17) showing heat flow (Q) against temperature (T). The eutectic (T_{eu}, first heating step, —) and excess (T_{ex}, second heating step, —) melting peaks are indicated, along with the glass transition (T_g, second cooling step, —). All other steps are omitted for clarity. b) Partial Tammann diagram of Phl/Cli showing the eutectic (●, —) and excess (×, ---) enthalpy of fusion (ΔH_{fus}) as a function of Phl mole fraction (f_{Phl}) in a mixture with Cli. Phl decomposes rather than melting, so the hypereutectic portion was not obtained. Least squares regression is applied to each linear portion of the plot and indicated as solid and dotted lines.

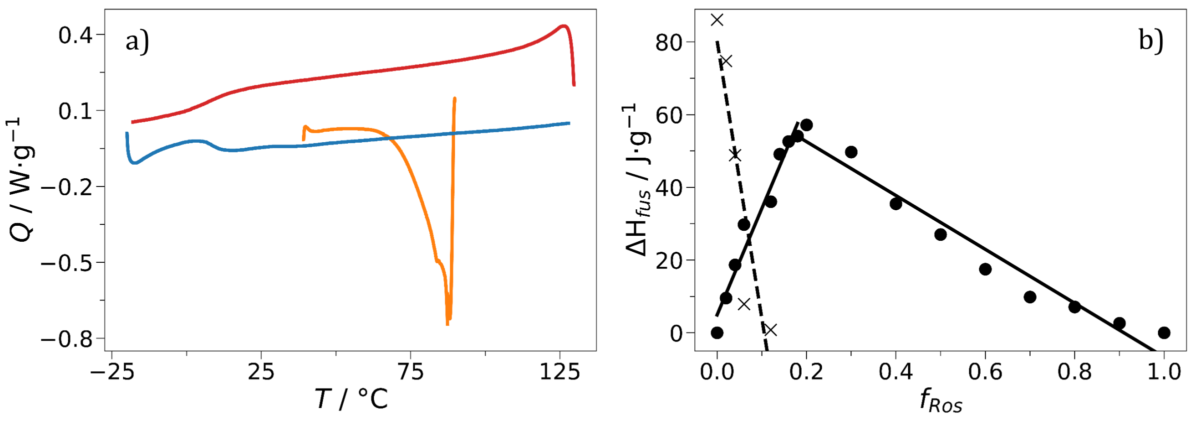

Figure S7. a) DSC thermogram of eutectic Ros/Cli (f_{Ros}= 0.17) showing heat flow (Q) against temperature (T). The eutectic (T_{eu}, first heating step, —) and excess (T_{ex}, second heating step, —) melting peaks are indicated, along with the glass transition (T_g, second cooling step, —). All other steps are omitted for clarity. b) Partial Tammann diagram of Ros/Cli showing the eutectic (●, —) and excess (×, ---) enthalpy of fusion (ΔH_{fus}) as a function of Ros mole fraction (f_{Ros}) in a mixture with Cli. Ros decomposes rather than melting, so the hypereutectic portion was not obtained. Least squares regression is applied to each linear portion of the plot and indicated as solid and dotted lines.

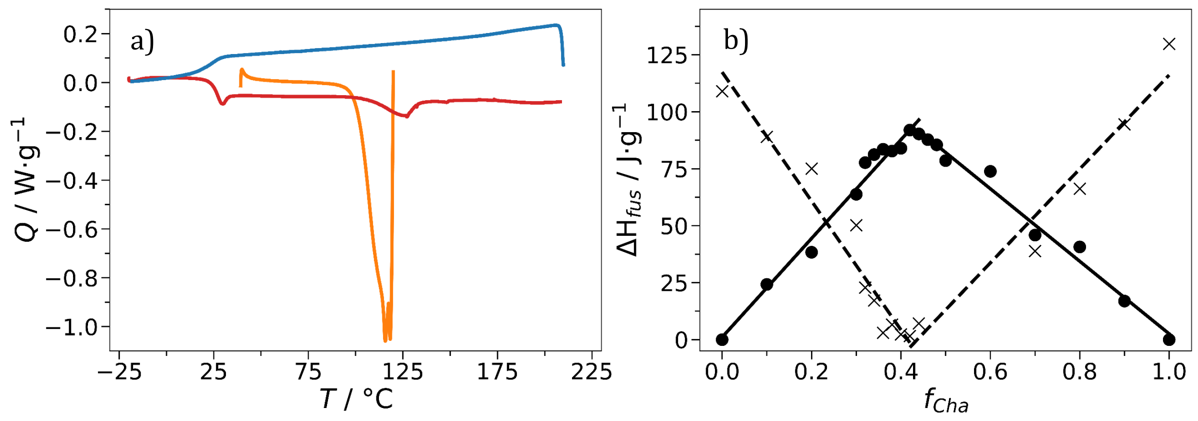

Figure S8. a) DSC thermogram of eutectic Cha/Bif (f_{Cha}= 0.42) showing heat flow (Q) against temperature (T). The eutectic (T_{eu}, first heating step, —) and excess (T_{ex}, second heating step, —) melting peaks are indicated, along with the glass transition (T_g, second cooling step, —). All other steps are omitted for clarity. b) Partial Tammann diagram of Cha/Bif showing the eutectic (●, —) and excess (×, ---) enthalpy of fusion (ΔH_{fus}) as a function of Cha mole fraction (f_{Cha}) in a mixture with Bif. Least squares regression is applied to each linear portion of the plot and indicated as solid and dotted lines.

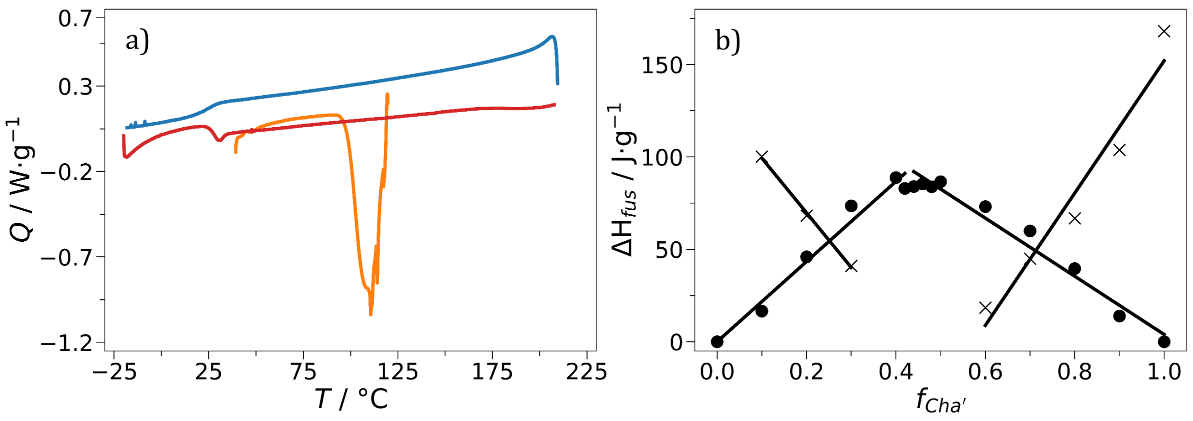

Figure S9. a) DSC thermogram of eutectic Cha'/Bif ($f_{Cha'}$= 0.42) showing heat flow (Q) against temperature (T). The eutectic (T_{eu}, first heating step, —) and excess (T_{ex}, second heating step, —) melting peaks are indicated, along with the glass transition (T_g, second cooling step, —). All other steps are omitted for clarity. b) Partial Tammann diagram of Cha'/Bif showing the eutectic (●, —) and excess (×, ---) enthalpy of fusion (ΔH_{fus}) as a function of Cha' mole fraction ($f_{Cha'}$) in a mixture with Bif. Least squares regression is applied to each linear portion of the plot and indicated as solid and dotted lines.

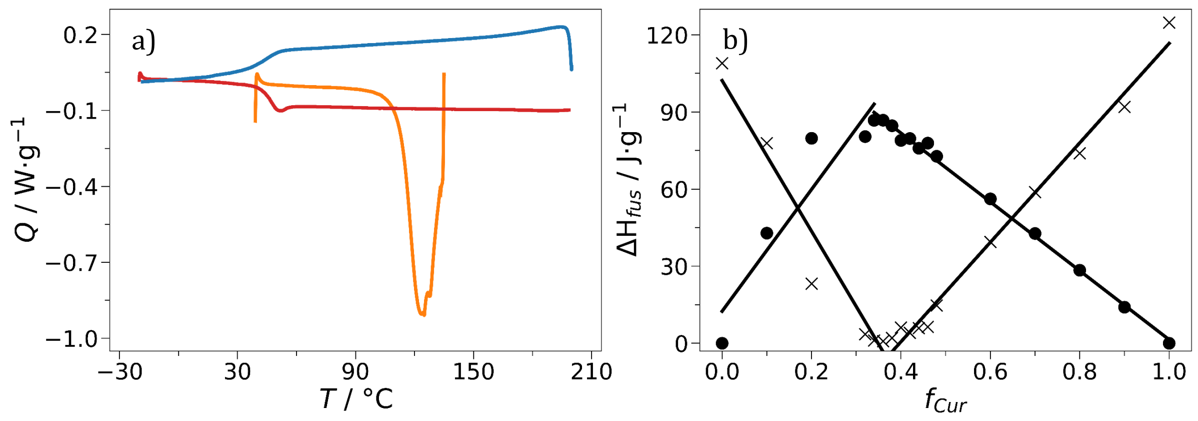

Figure S10. a) DSC thermogram of eutectic Cur/Bif (f_{Cur}= 0.35) showing heat flow (Q) against temperature (T). The eutectic (T_{eu}, first heating step, —) and excess (T_{ex}, second heating step, —) melting peaks are indicated, along with the glass transition (T_g, second cooling step, —). All other steps are omitted for clarity. b) Partial Tammann diagram of Cur/Bif showing the eutectic (●, —) and excess (×, ---) enthalpy of fusion (ΔH_{fus}) as a function of Cur mole fraction (f_{Cur}) in a mixture with Bif. Least squares regression is applied to each linear portion of the plot and indicated as solid and dotted lines.

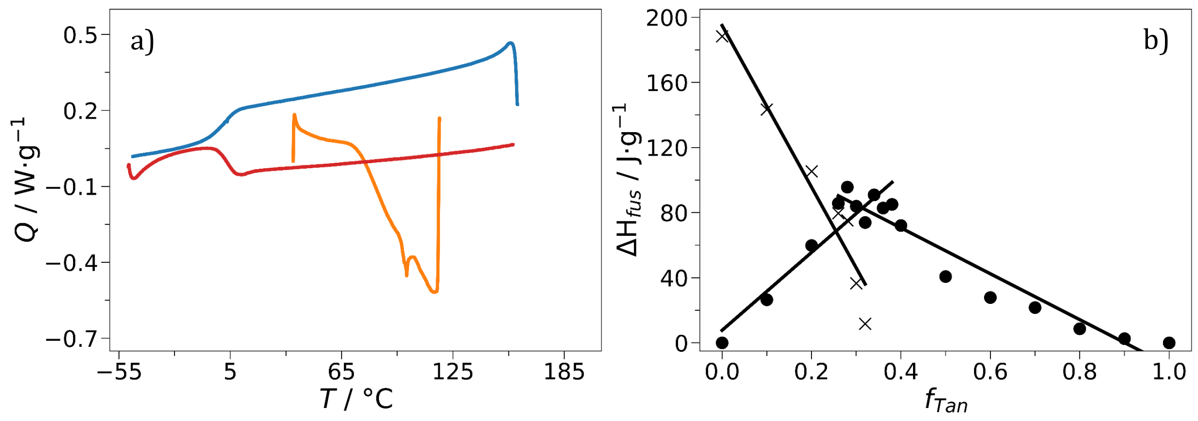

Figure S11. a) DSC thermogram of eutectic Tan/Nic (f_{Tan}= 0.31) showing heat flow (Q) against temperature (T). The eutectic (T_{eu}, first heating step, —) and excess (T_{ex}, second heating step, —) melting peaks are indicated, along with the glass transition (T_g, second cooling step, —). All other steps are omitted for clarity. b) Partial Tammann diagram of Tan/Nic showing the eutectic (●, —) and excess (×, ---) enthalpy of fusion (ΔH_{fus}) as a function of Tan mole fraction (f_{Tan}) in a mixture with Nic. Tan decomposes rather than melting, so the hypereutectic portion was not obtained. Least squares regression is applied to each linear portion of the plot and indicated as solid and dotted lines.

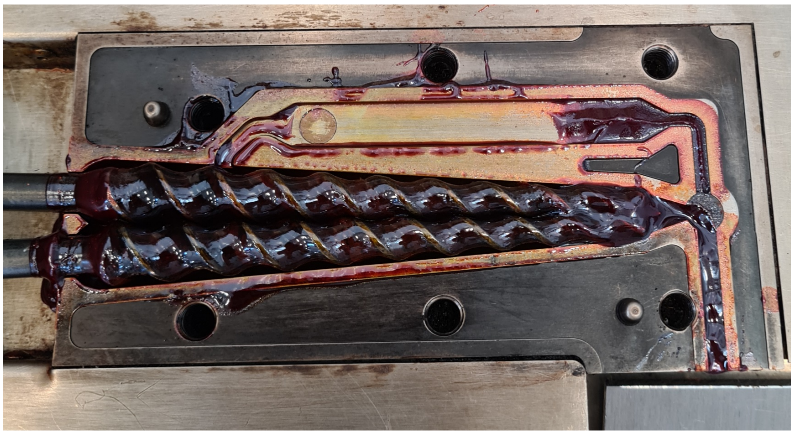

Figure S12. Cur/Bif mini-extrusion pilot testing. The efficient mixing and heating enables facile eutectic formation.

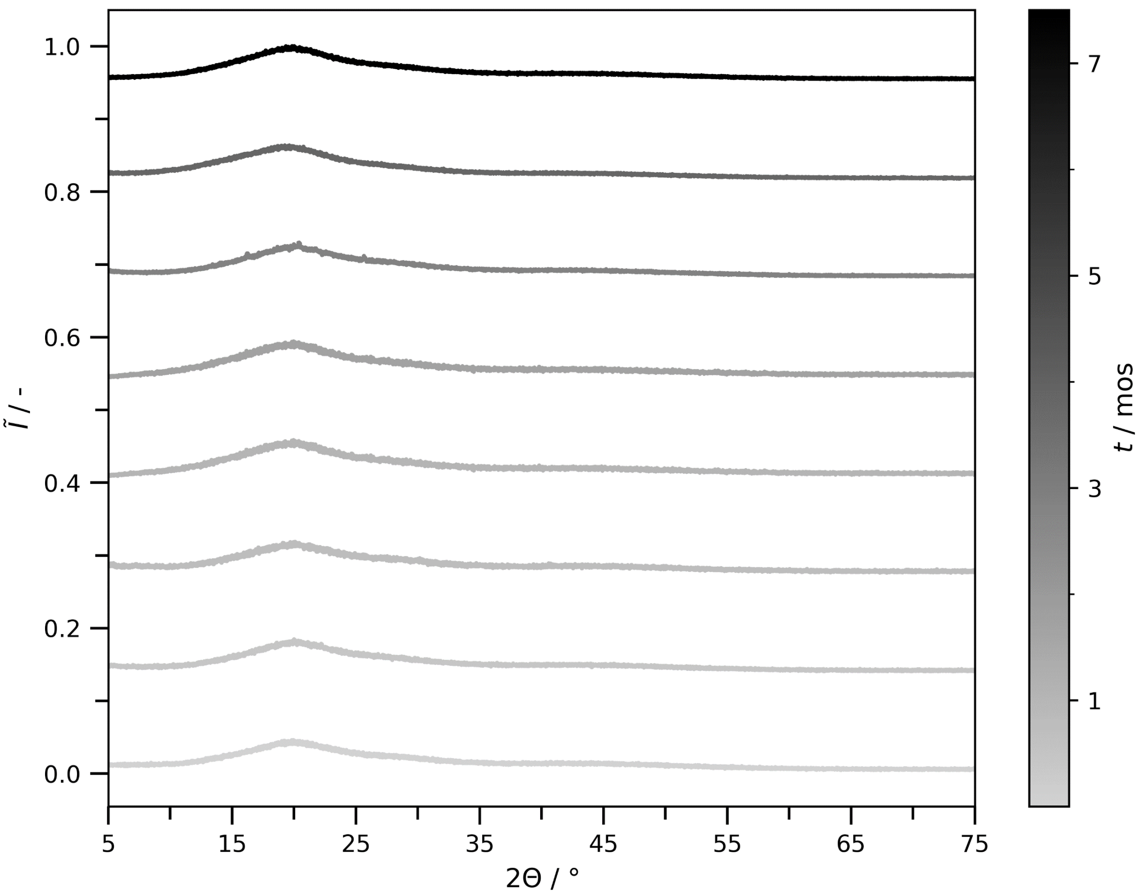

Figure S13. Cur/Cli XRD patterns over 7 months (mos) demonstrating good stability of the broad amorphous phase against crystallization. Intensity is Min-Max normalized (Ĩ) so values lie between 0 and 1. Diffraction patterns were recorded in the 2θ range 5-75° (step size 0.013°) with each experiment totaling 30 or 60 min.

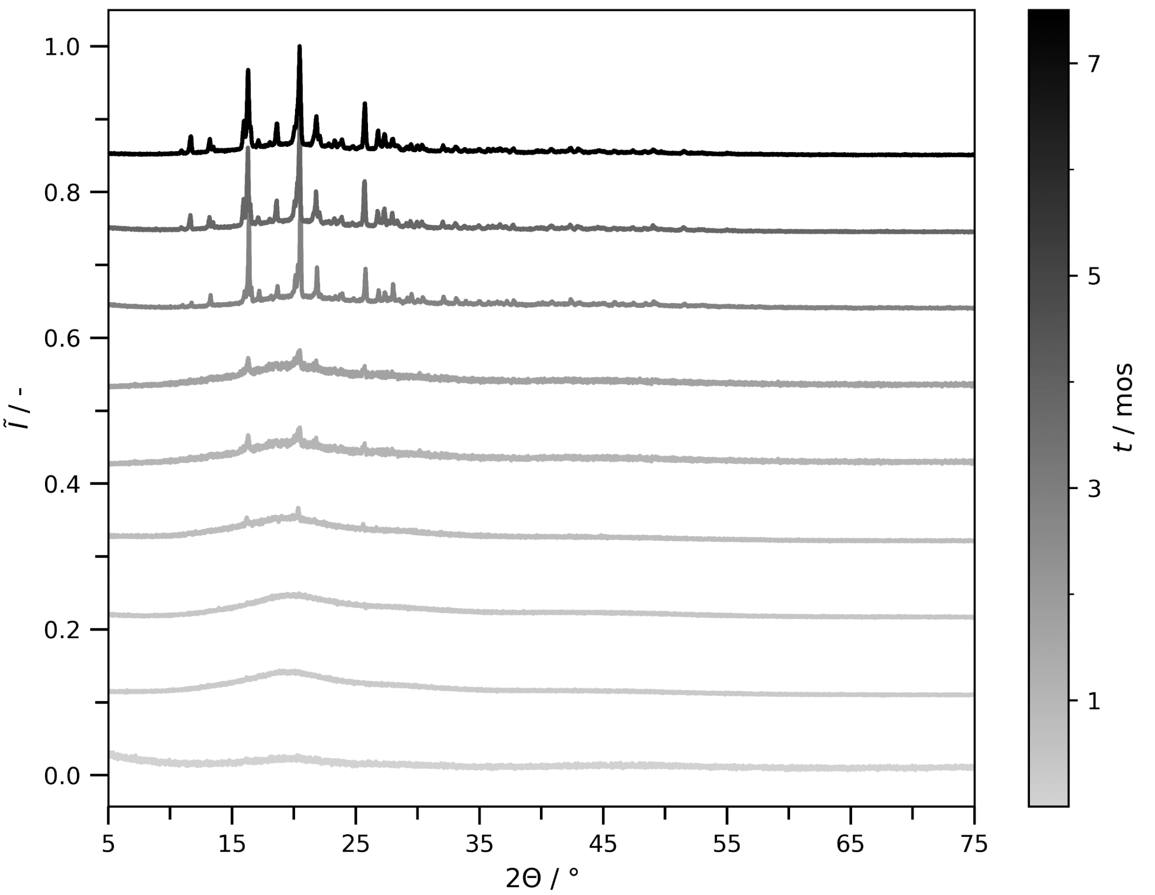

Figure S14. Phl/Cli XRD patterns over 7 months (mos) demonstrating reasonable stability of the broad amorphous phase against crystallization. Crystalline material begins to emerge after 3 weeks, although growth is slow, and some amorphous character is retained even at 7 months. Intensity is Min-Max normalized (Ĩ) so values lie between 0 and 1. Diffraction patterns were recorded in the 2θ range 5-75° (step size 0.013°) with each experiment totaling 30 or 60 min.

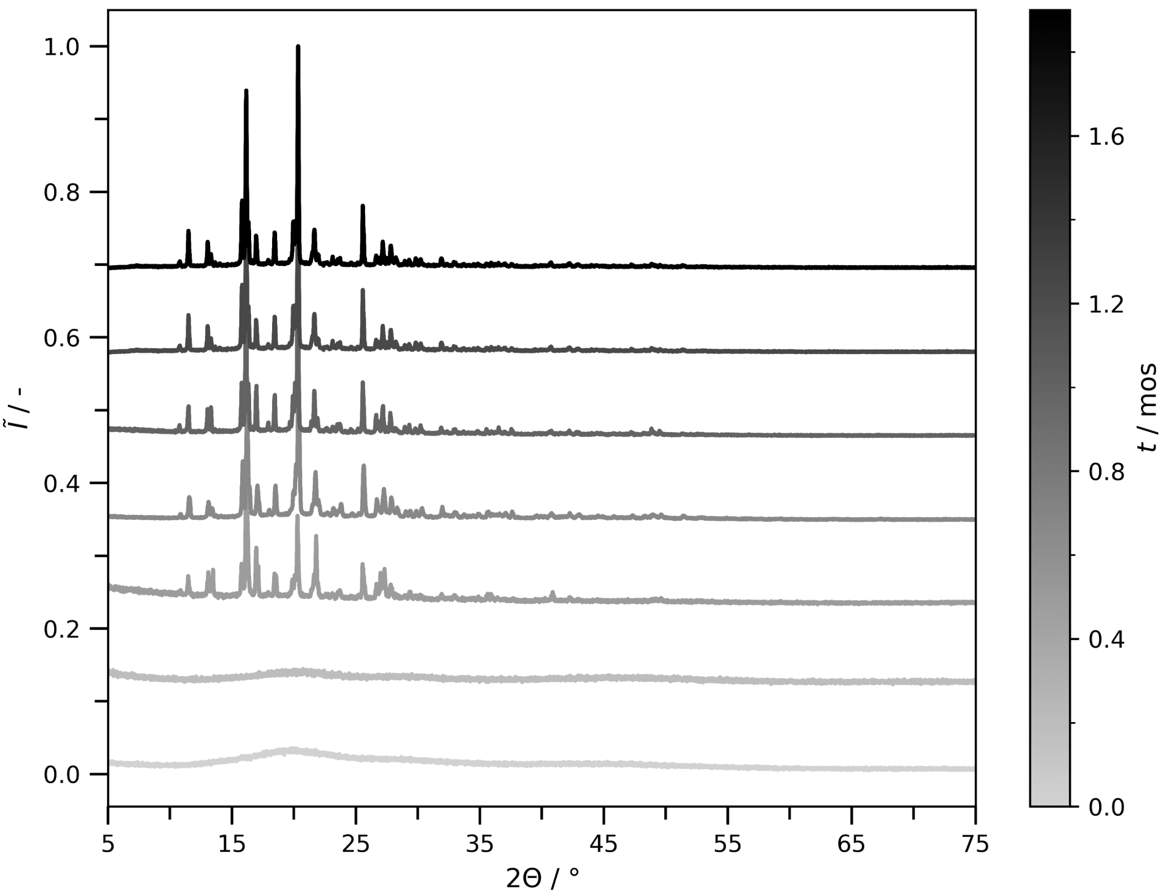

Figure S15. Ros/Cli XRD patterns over 2 months (mos) demonstrating poor stability of the broad amorphous phase against crystallization. Crystalline material begins to emerge after 2 weeks, and amorphous character is lost entirely after 3 weeks. Intensity is Min-Max normalized (Ĩ) so values lie between 0 and 1. Diffraction patterns were recorded in the 2θ range 5-75° (step size 0.013°) with each experiment totaling 30 or 60 min.

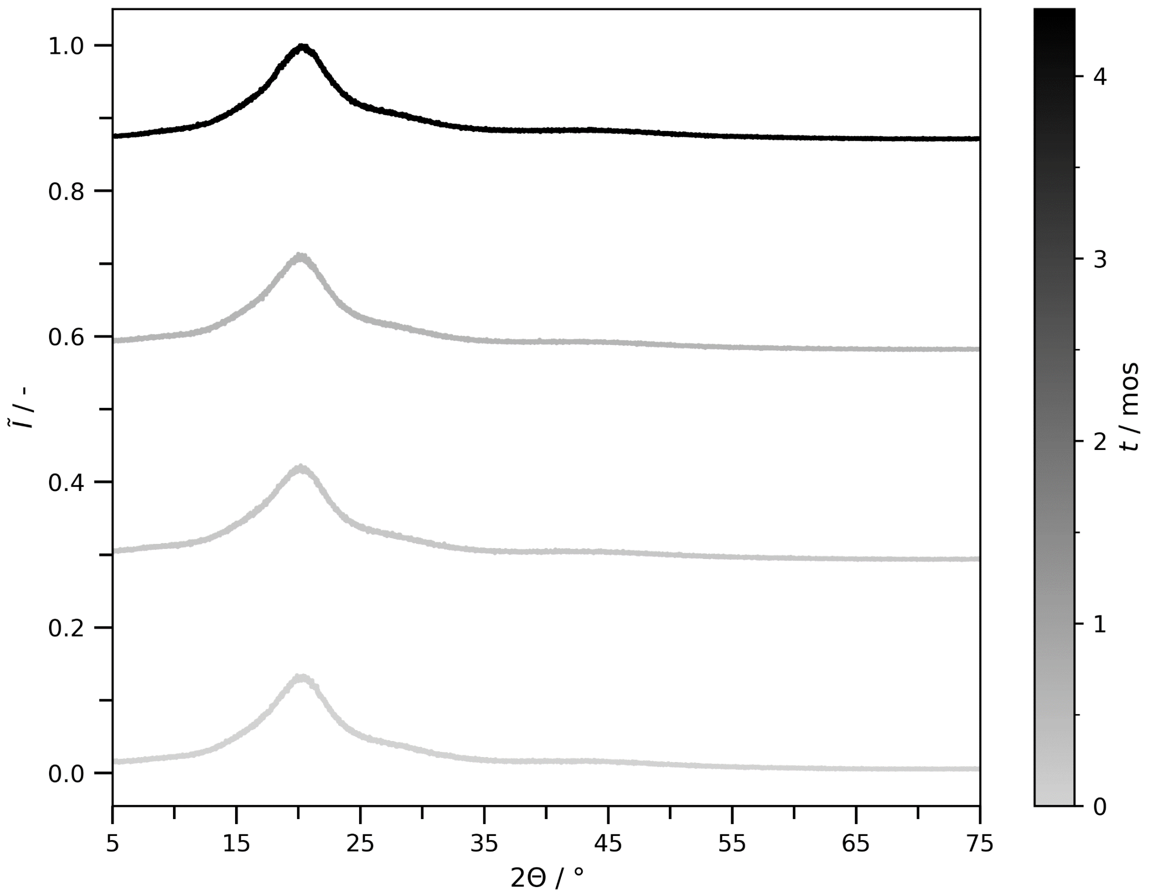

Figure S16. Cha'/Bif XRD patterns over 4 months (mos) demonstrating good stability of the broad amorphous phase against crystallization. Intensity is Min-Max normalized (Ĩ) so values lie between 0 and 1. Diffraction patterns were recorded in the 2θ range 5-75° (step size 0.013°) with each experiment totaling 30 or 60 min.

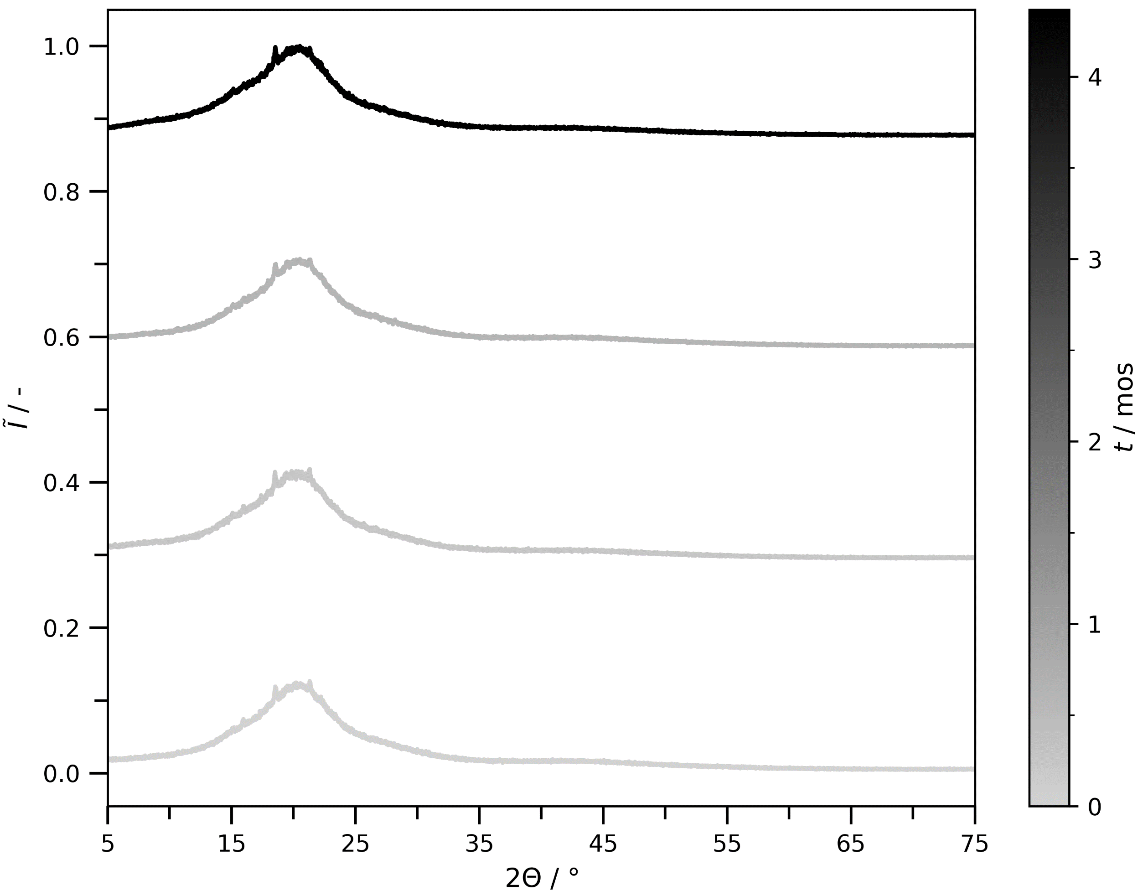

Figure S17. Cur/Bif XRD patterns over 4 months (mos) demonstrating good stability of the broad amorphous phase against crystallization. Small crystalline peaks are present throughout but do not grow to any noticeable extent. These are likely caused by the residual 'orange powder' discussed in the main text. Intensity is Min-Max normalized (Ĩ) so values lie between 0 and 1. Diffraction patterns were recorded in the 2θ range 5-75° (step size 0.013°) with each experiment totaling 30 or 60 min.

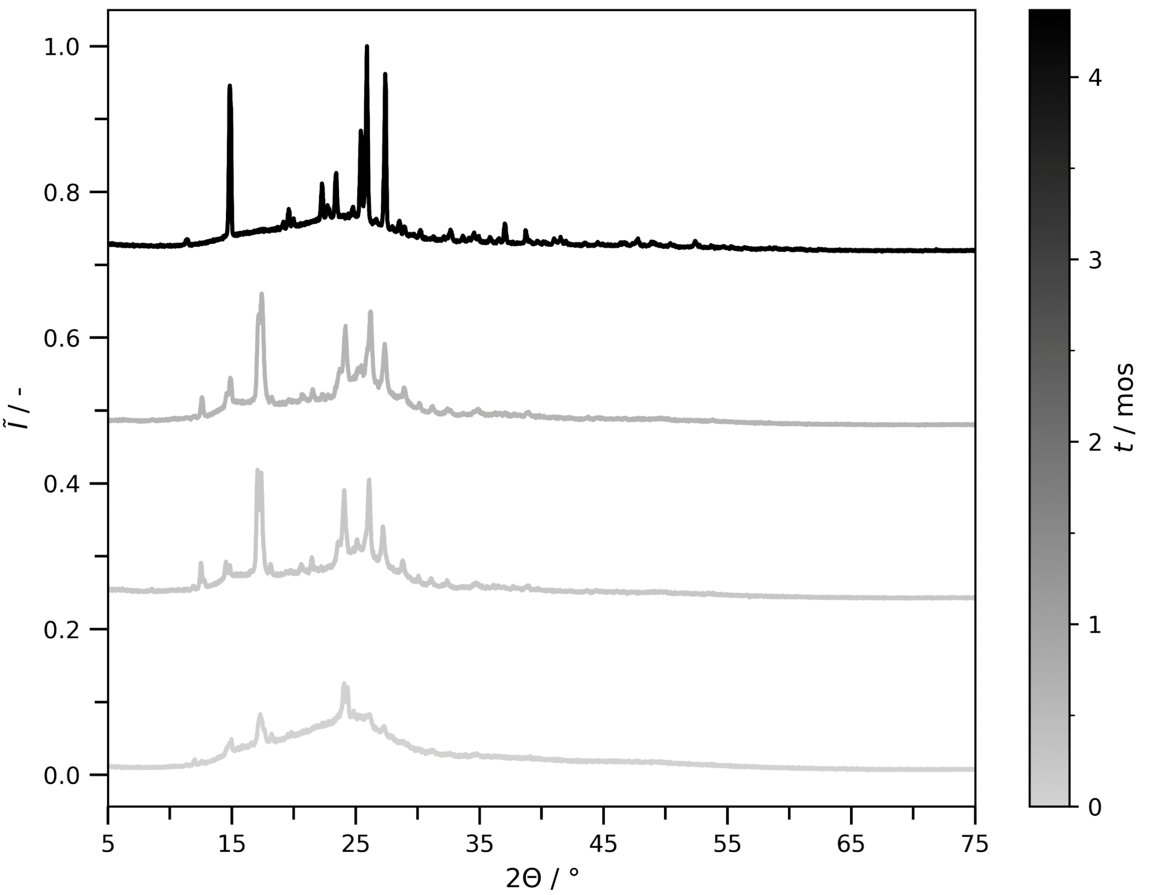

Figure S18. Tan/Nic XRD patterns over 4 months (mos) demonstrating poor stability of the broad amorphous phase against crystallization. Small crystalline peaks are present from t = 0. These are likely caused by sublimation of Nic during sample preparation which shifts the composition away from that of the eutectic. Intensity is Min-Max normalized (Ĩ) so values lie between 0 and 1. Diffraction patterns were recorded in the 2θ range 5-75° (step size 0.013°) with each experiment totaling 30 or 60 min.

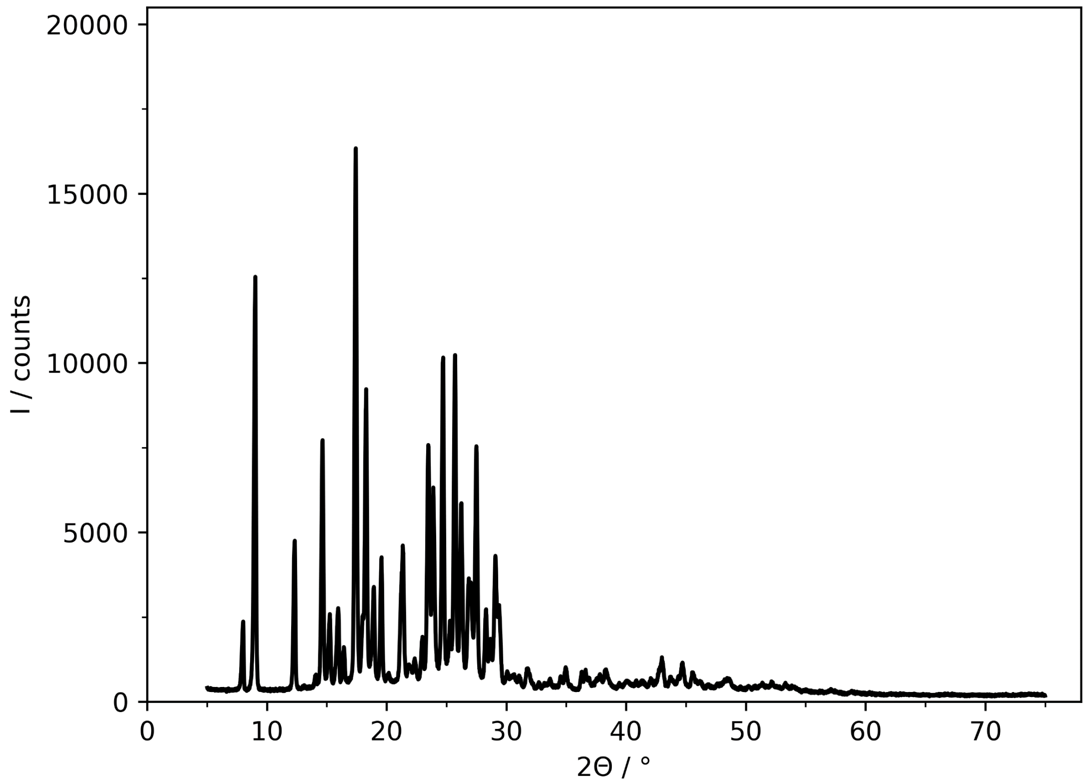

Figure S19. XRD of curcumin (Cur). The diffraction pattern was recorded in the 2θ range 5-75° (step size 0.013°) over 30 min.

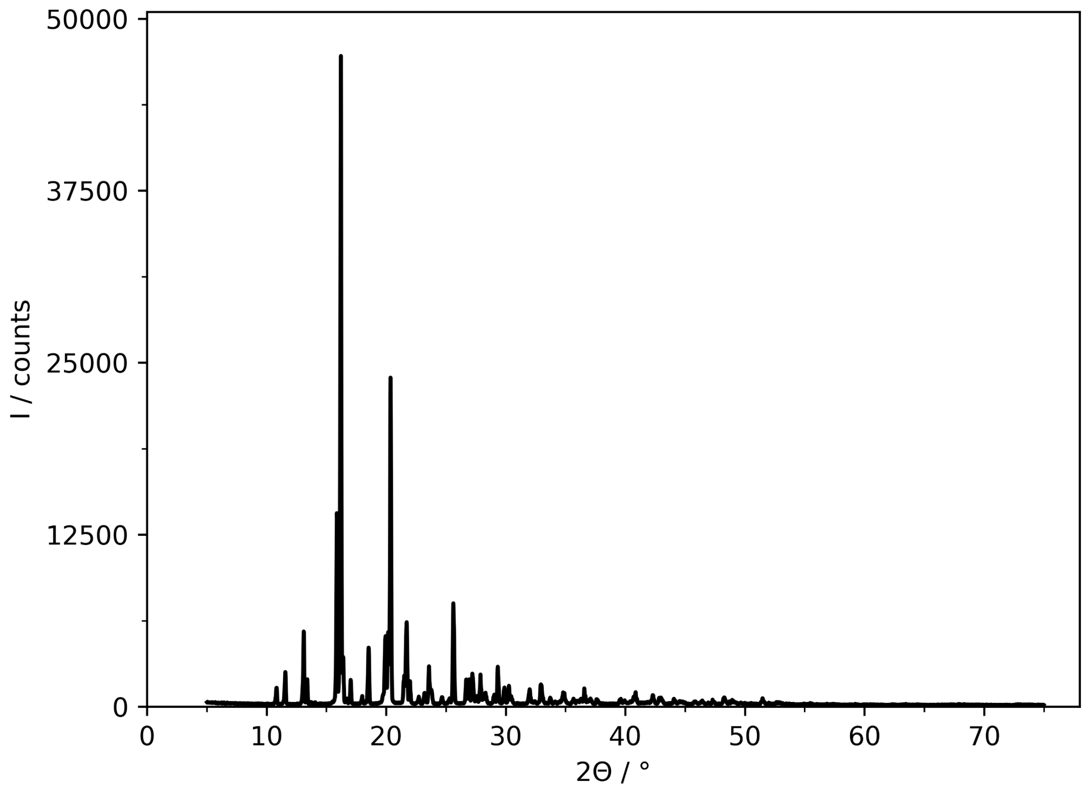

Figure S20. XRD of climbazole (Cli). The diffraction pattern was recorded in the 2θ range 5-75° (step size 0.013°) over 30 min.

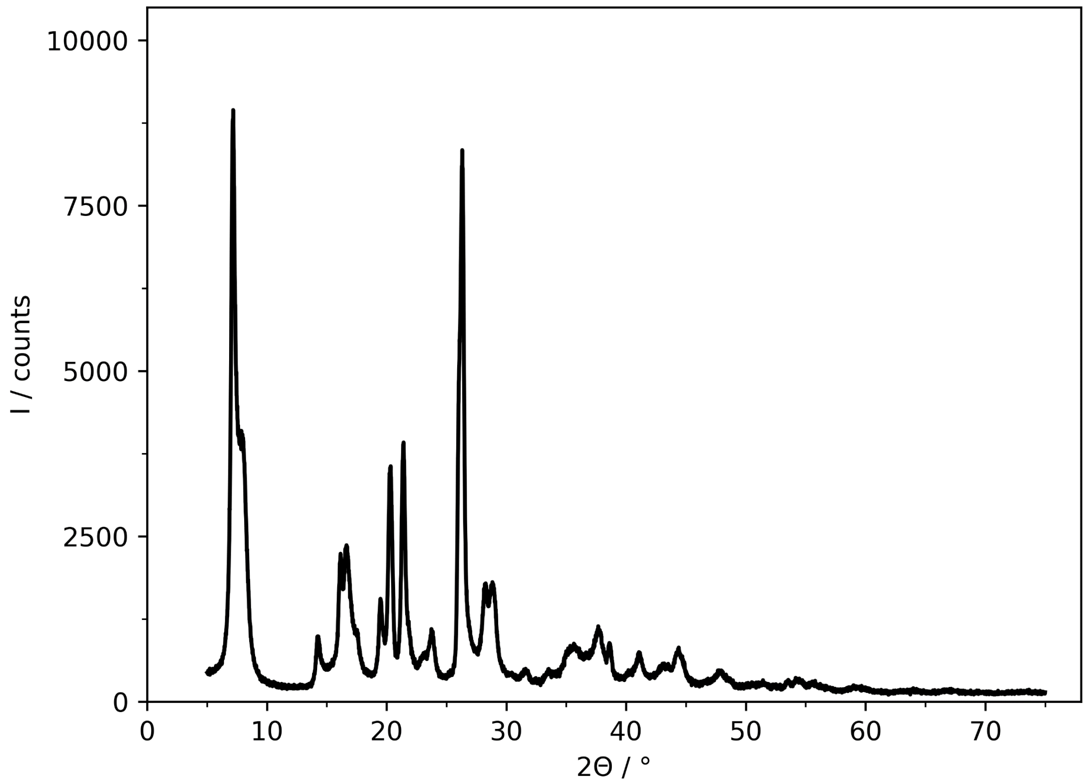

Figure S21. XRD of phloroglucide (Phl). The diffraction pattern was recorded in the 2θ range 5-75° (step size 0.013°) over 30 min.

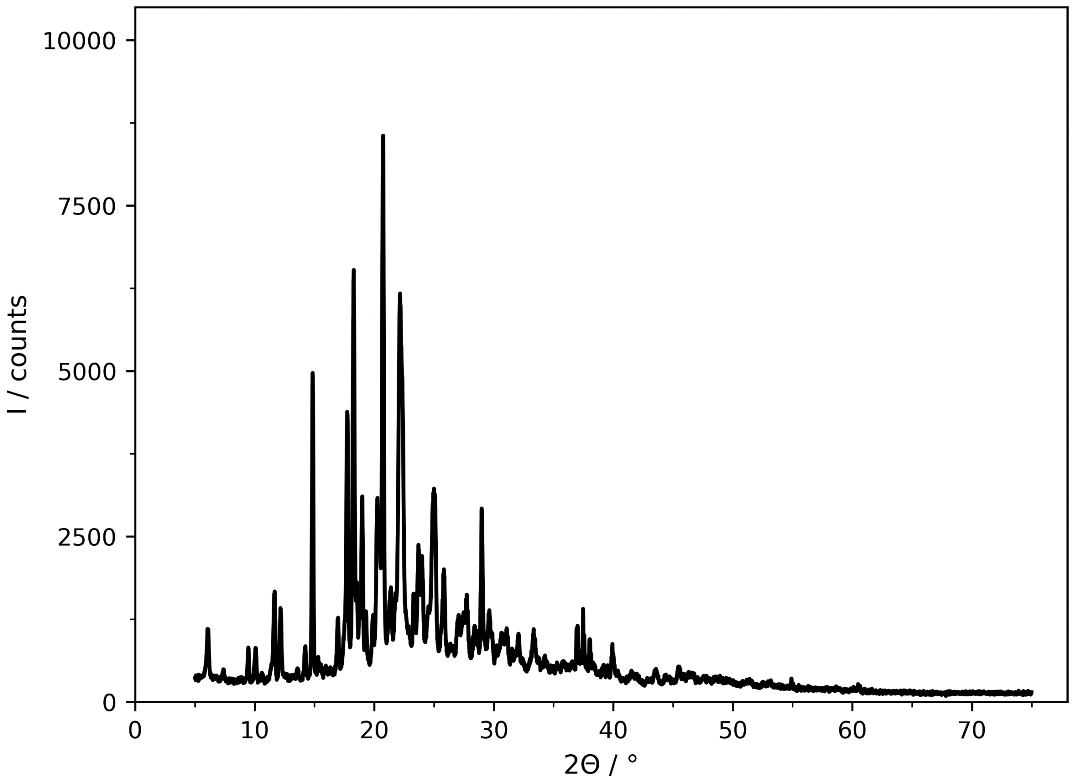

Figure S22. XRD of rosolic acid (Ros). The diffraction pattern was recorded in the 2θ range 5-75° (step size 0.013°) over 30 min.

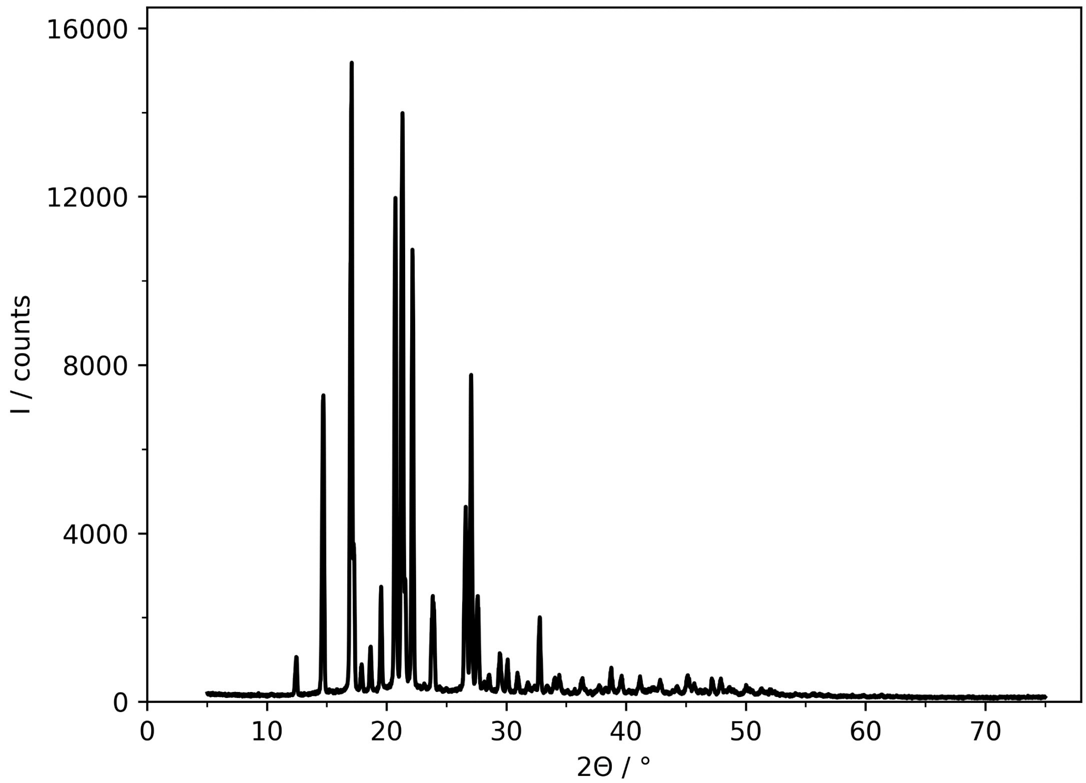

Figure S23. XRD of 4-hydroxychalcone (Cha). The diffraction pattern was recorded in the 2θ range 5-75° (step size 0.013°) over 30 min.

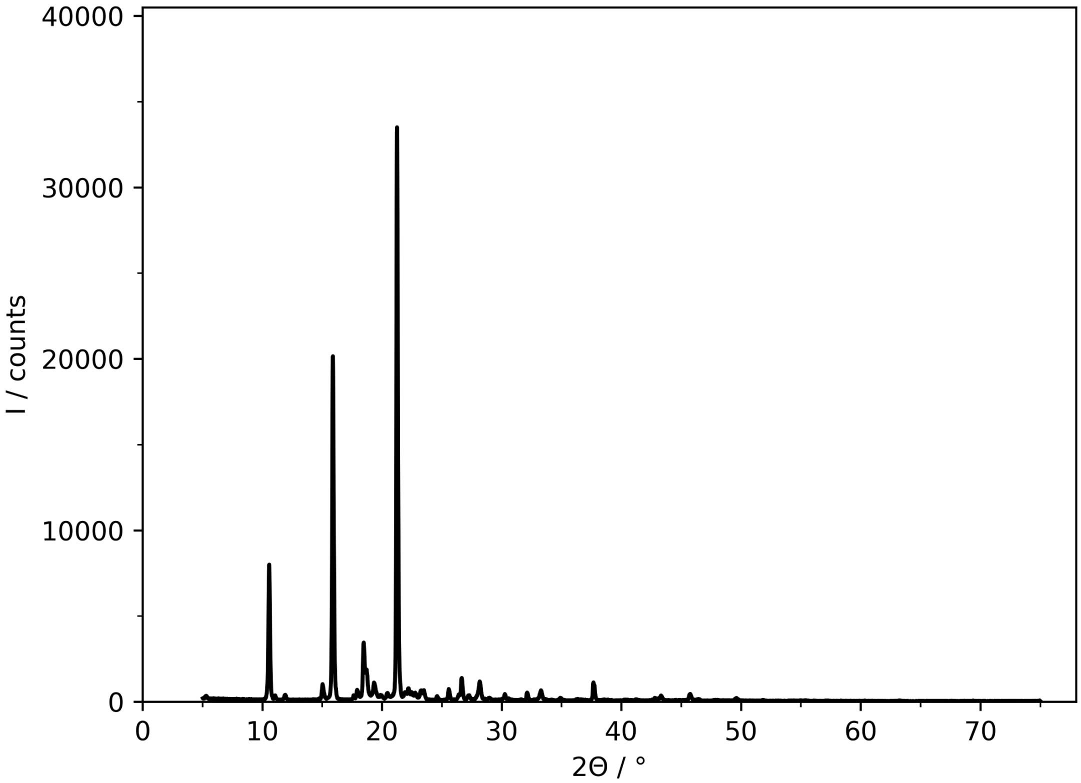

Figure S24. XRD of bifonazole (Bif). The diffraction pattern was recorded in the 2θ range 5-75° (step size 0.013°) over 30 min.

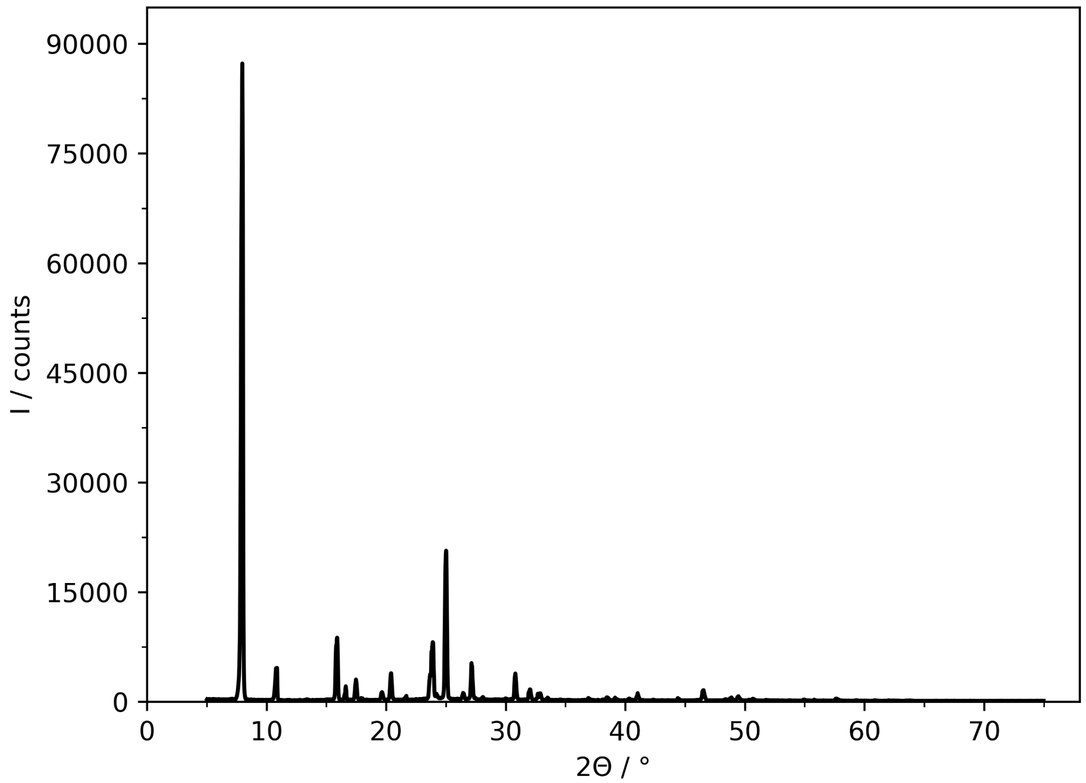

Figure S25. XRD of 4-Hydroxychalcone (Cha'). The diffraction pattern was recorded in the 2θ range 5-75° (step size 0.013°) over 30 min.

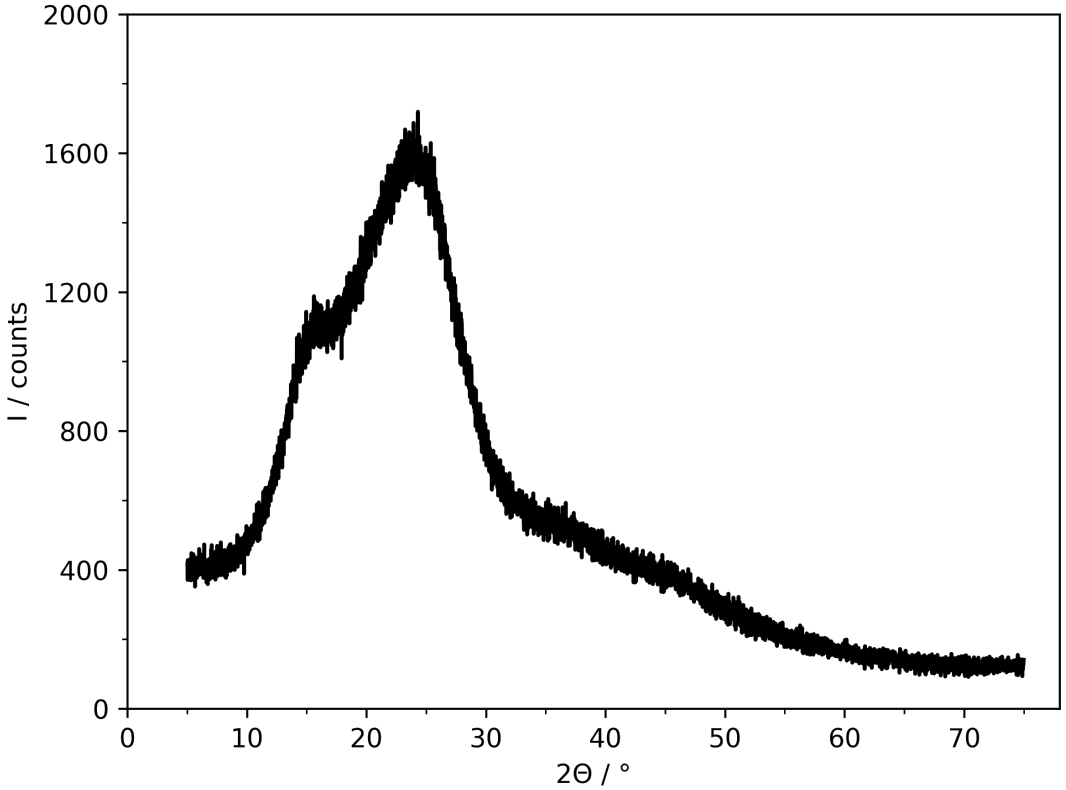

Figure S26. XRD of Tannic acid (Tan). The diffraction pattern was recorded in the 2θ range 5-75° (step size 0.013°) over 30 min.

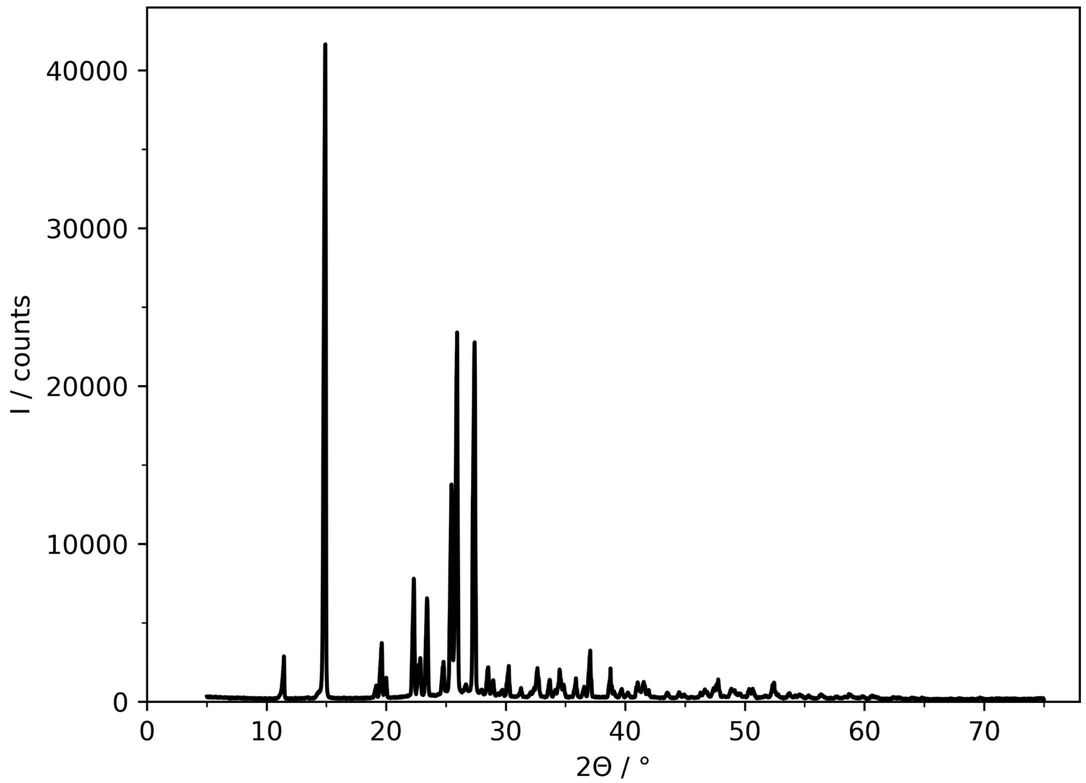

Figure S27. XRD of Nicotinamide (Nic). The diffraction pattern was recorded in the 2θ range 5-75° (step size 0.013°) over 30 min.

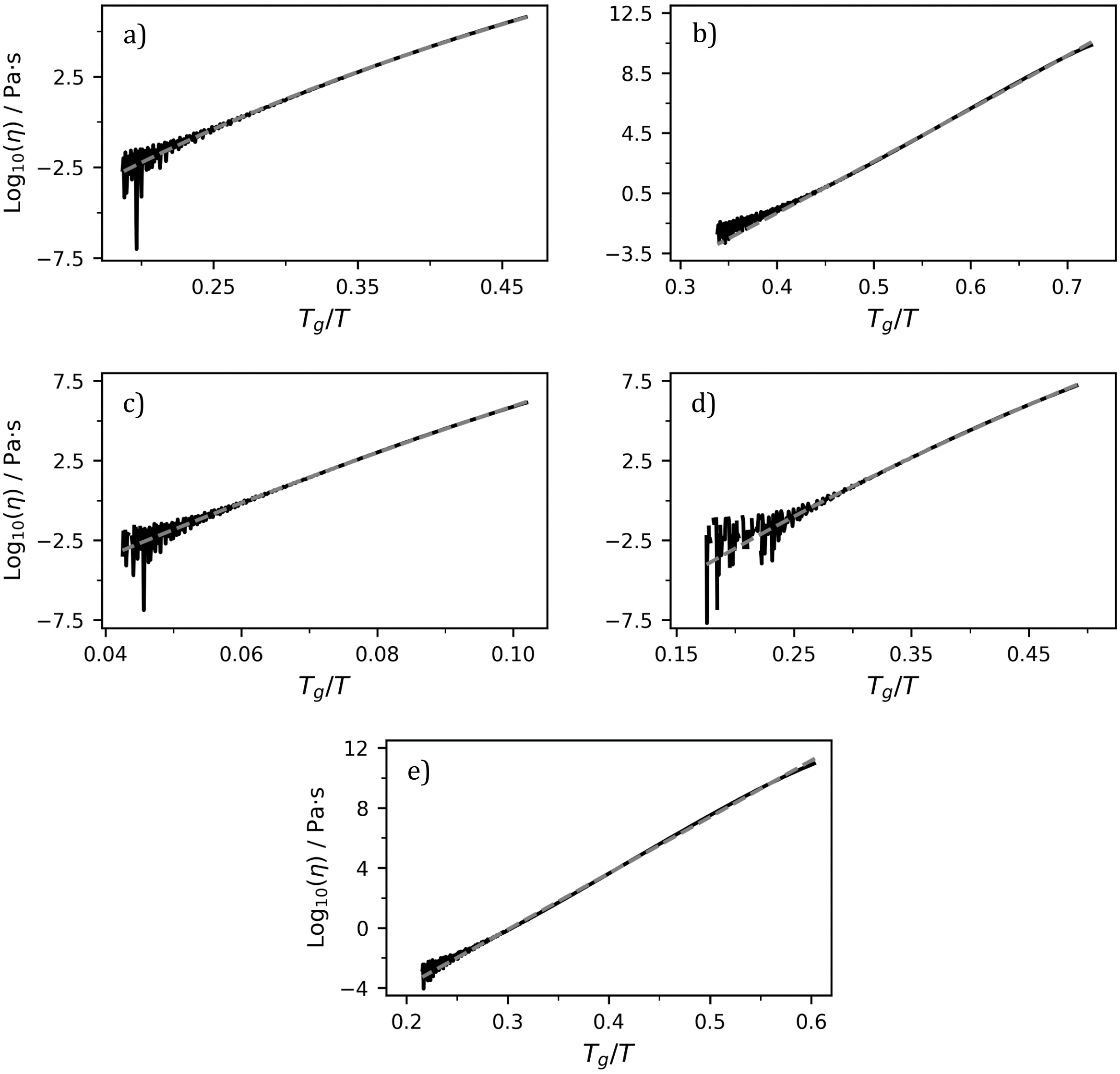

Figure S28. Angell plots showing the dependence of viscosity (η) on temperature (T) scaled by T_g for a) Ros/Cli, b) Cur/Bif, c) Phl/Cli, d) Cur/Cli and e) Cha'/Bif.[1] A VFT equation fit (---) of the data (—) is overlayed. The ramp rate (2 °C·min^{-1}), rotational velocity (1 rad·s^{-1}) and parallel plate gap (650 μm) were constant for all experiments.

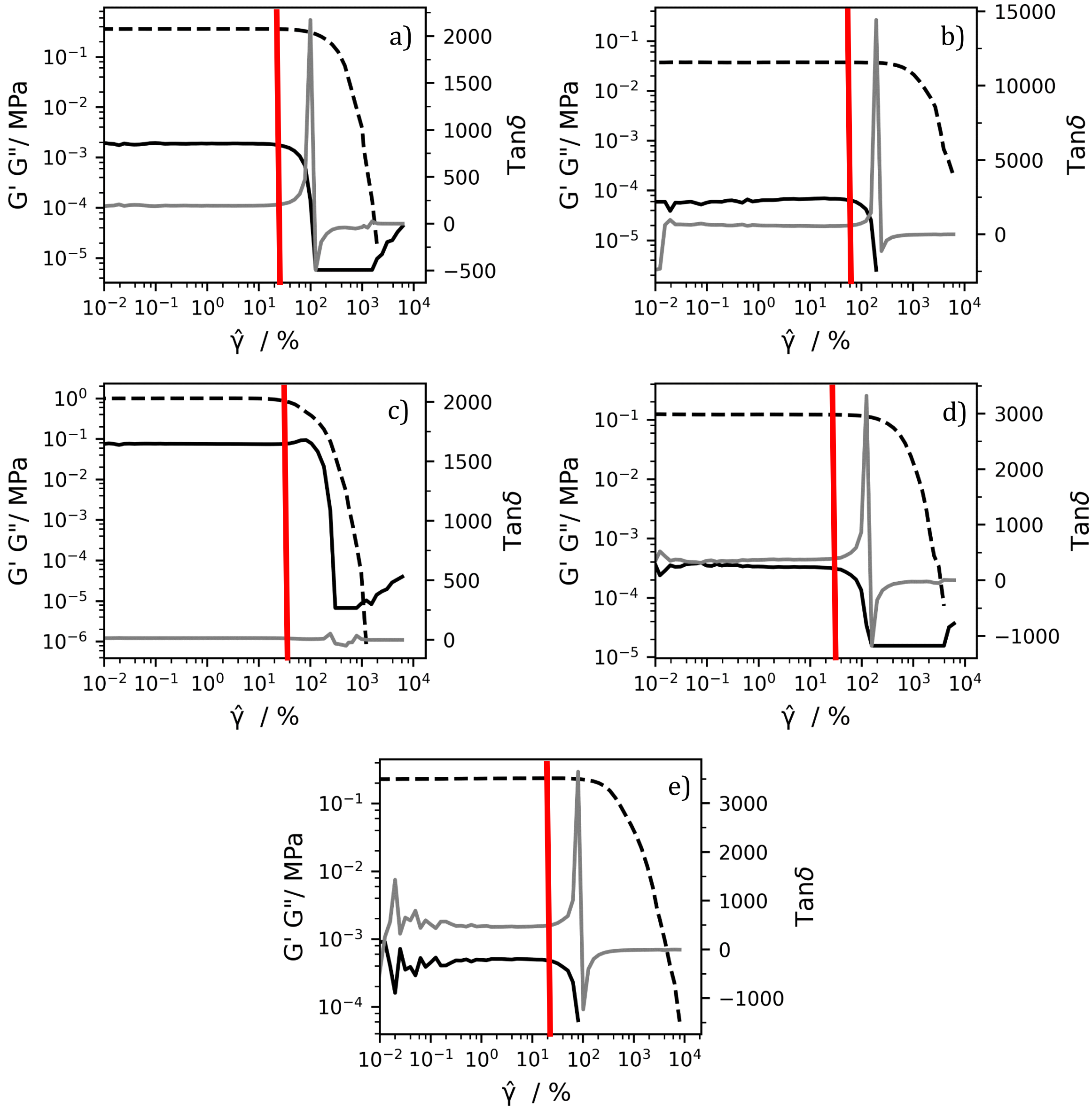

Figure S29. Amplitude sweeps showing the dependence of the storage modulus (G', —), loss modulus (G'', ---) and tanδ (—) on oscillation strain ($\hat{\gamma}$) for a) Ros/Cli, b) Cur/Bif, c) Phl/Cli, d) Cur/Cli and e) Cha'/Bif. The upper bound of the linear viscoelastic region (LVER) is indicated by a vertical red line. Data below $\hat{\gamma}$ = 10^{-2} % becomes noisy due to sensitivity limitations of the instrument and is omitted for clarity. Above the LVER each sample exhibits shear thinning, as indicated by a steady drop in G' and G''. More details can be found in the main text. The oscillation amplitude (2.0 × 10^{-8} to 10.0 rad), angular frequency (5 or 10 rad·s^{-1}) and parallel plate gap (650 μm) were constant for all experiments.

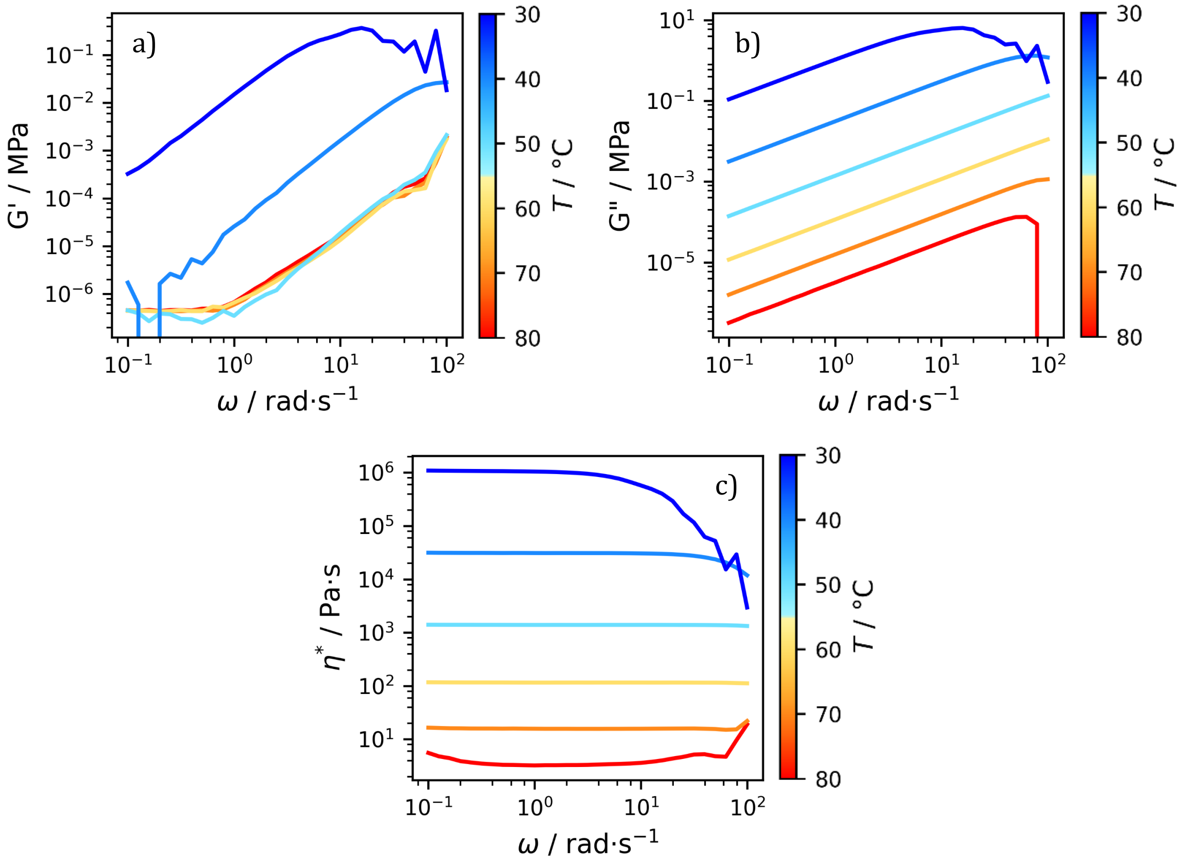

Figure S30. Oscillatory frequency sweeps of Cur/Cli showing the dependence of a) storage modulus (*G'*), b) loss modulus (*G"*) and c) complex viscosity (η^*) on angular frequency (ω) and temperature (*T*). Shear thinning behavior is exhibited at low *T* and high ω. The frequency range (0.1 – 100 rad·s^{-1}) was constant for all temperatures. See **Table S4** for details on oscillation amplitude and displacement parameters.

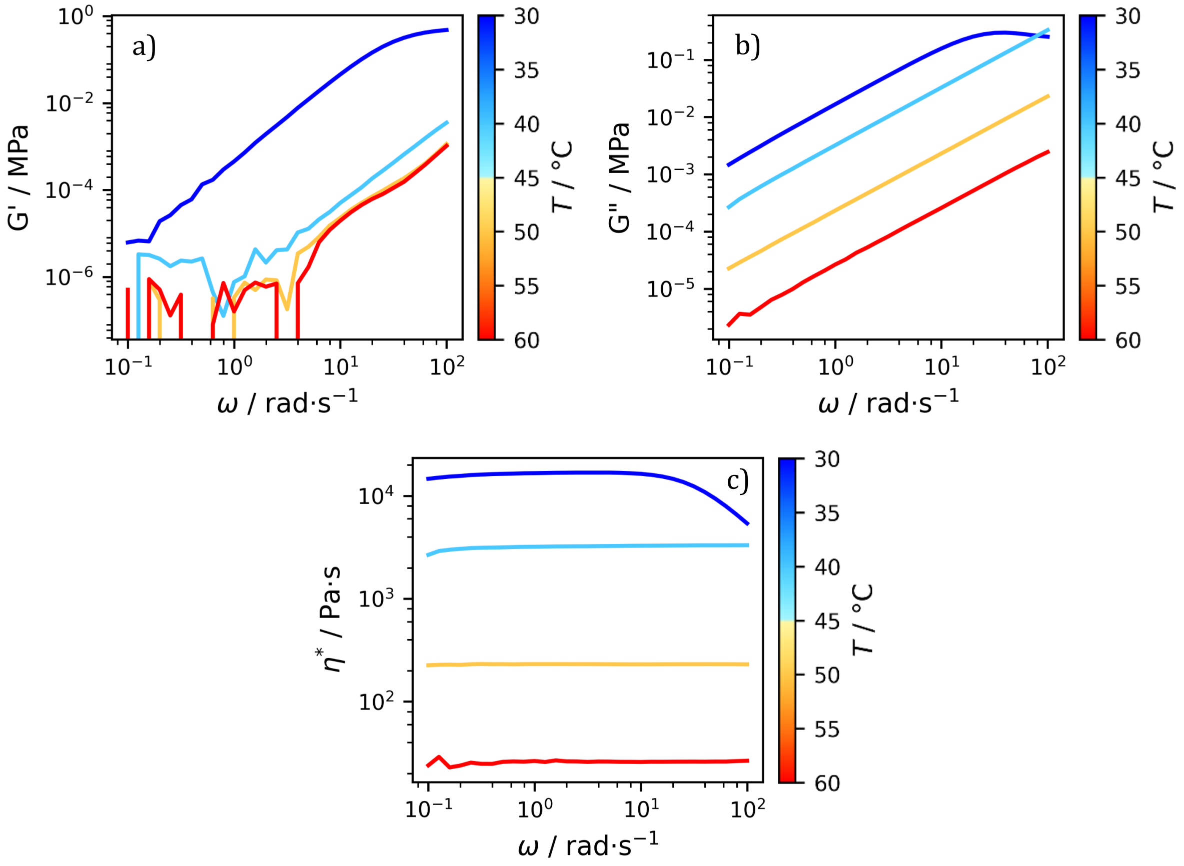

Figure S31. Oscillatory frequency sweeps of Phl/Cli showing the dependence of a) storage modulus (G'), b) loss modulus (G'') and c) complex viscosity (η^*) on angular frequency (ω) and temperature (T). Shear thinning behavior is exhibited at low T and high ω. See **Table S4** for details on oscillation amplitude and displacement parameters.

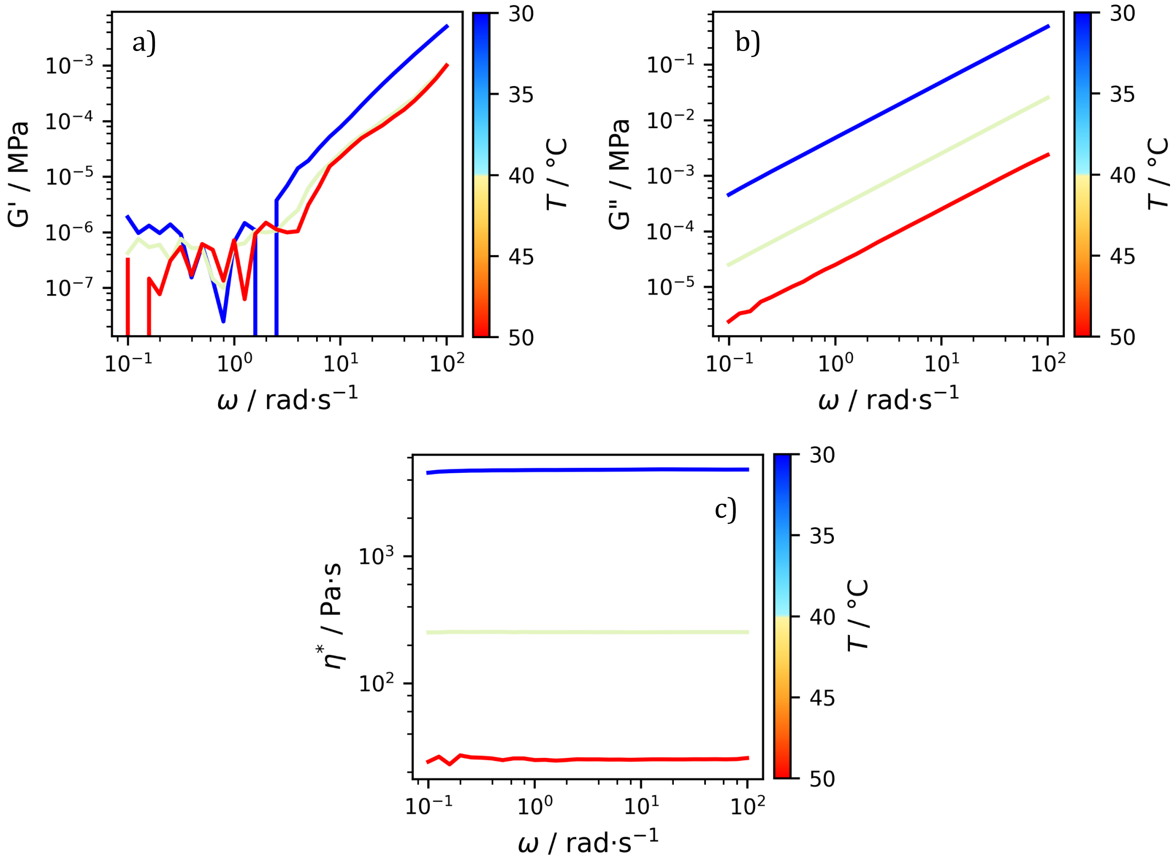

Figure S32. Oscillatory frequency sweeps of Ros/Cli showing the dependence of a) storage modulus (*G'*), b) loss modulus (*G"*) and c) complex viscosity (η^*) on angular frequency (ω) and temperature (*T*). See **Table S4** for details on oscillation amplitude and displacement parameters.

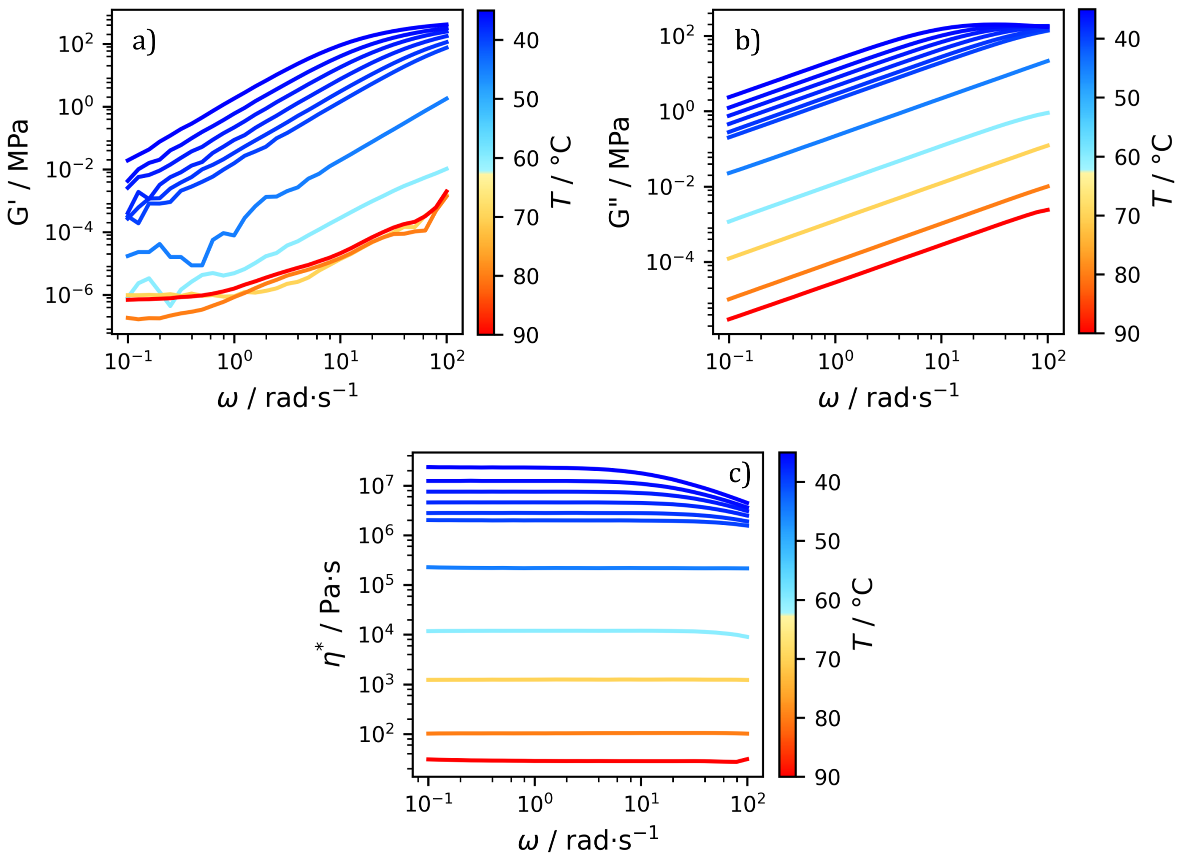

Figure S33. Oscillatory frequency sweeps of Cha/Bif showing the dependence of a) storage modulus (*G'*), b) loss modulus (*G"*) and c) complex viscosity (η^*) on angular frequency (ω) and temperature (*T*). Shear thinning behavior is exhibited at low *T* and high ω. See **Table S4** for details on oscillation amplitude and displacement parameters.

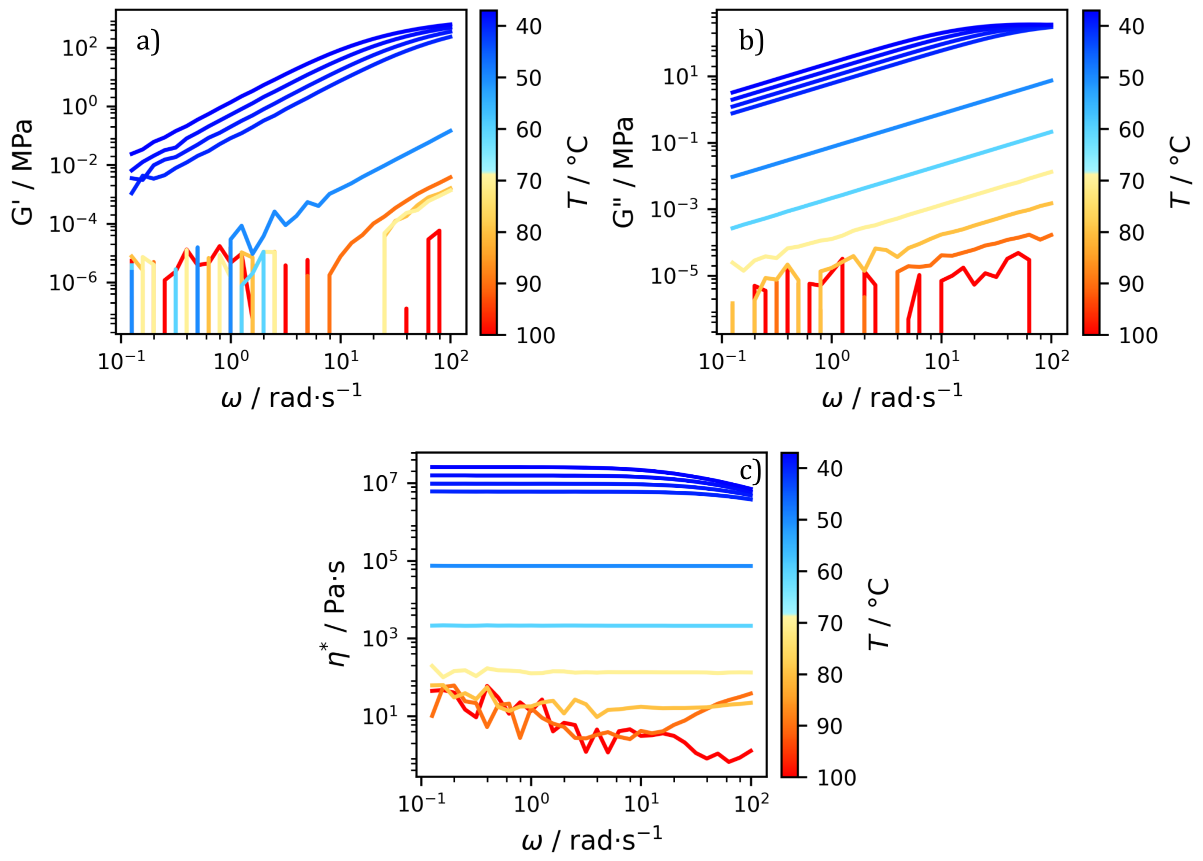

Figure S34. Oscillatory frequency sweeps of Cha'/Bif showing the dependence of a) storage modulus (*G'*), b) loss modulus (*G"*) and c) complex viscosity (η^*) on angular frequency (ω) and temperature (*T*). Shear thinning behavior is exhibited at low *T* and high ω. See **Table S4** for details on oscillation amplitude and displacement parameters.

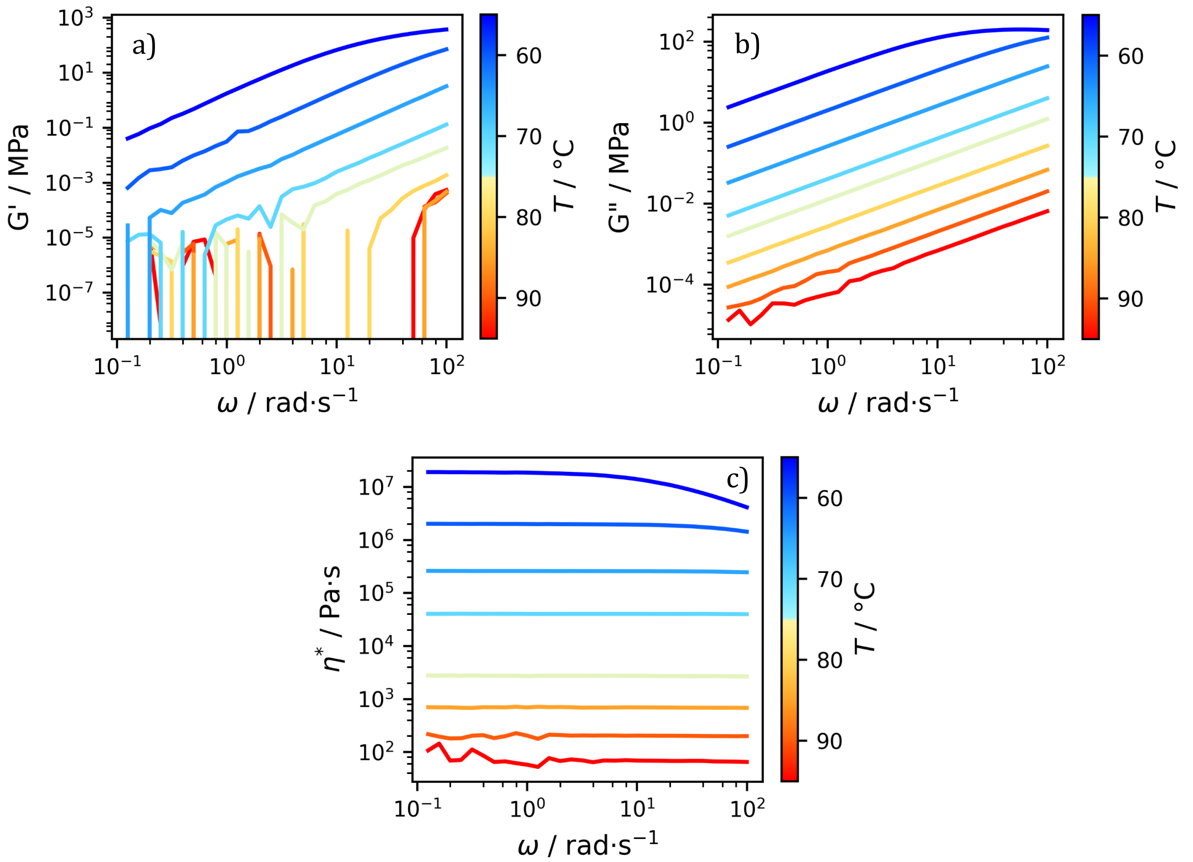

Figure S35. Oscillatory frequency sweeps of Cur/Bif showing the dependence of a) storage modulus (G'), b) loss modulus (G'') and c) complex viscosity (η^*) on angular frequency (ω) and temperature (T). Shear thinning behavior is exhibited at low T and high ω. See **Table S4** for details on oscillation amplitude and displacement parameters.

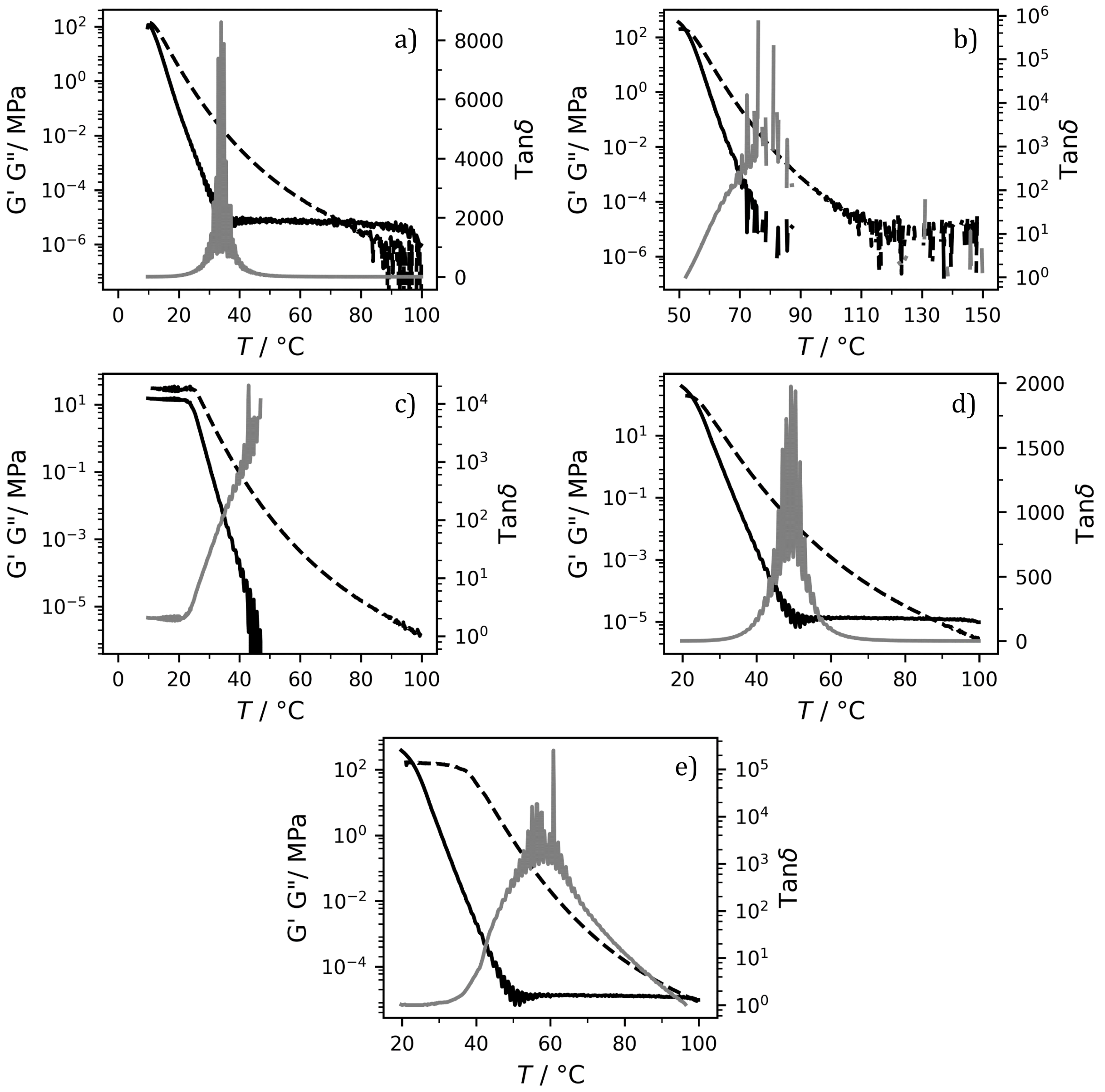

Figure S36. Oscillatory sweeps showing the dependence of the storage modulus (G', —), loss modulus (G'', ---) and tanδ (—) on temperature for a) Ros/Cli, b) Cur/Bif, c) Phl/Cli, d) Cur/Cli and e) Cha'/Bif. The cross-over of G' and G'' at low T indicates the T_g. Data below this point becomes unreliable due to instrument limitations associated with shearing such viscous media. The ramp rate (2 °C·min^{-1}) was constant for all experiments. See **Table S5** for angular frequency and displacement parameters.

PLSR calibration solution concentrations:

Solution number	[Cha] / mg·mL^{-1}	[Bif] / mg·mL^{-1}
1	1.525	5.996
2	3.075	3.587
3	4.485	1.905
4	6.152	4.998
5	7.475	2.484
6	0.501	2.934
7	1.060	0.623
8	5.269	7.271
9	7.951	1.751
10	8.961	4.165
11	0	1.936
12	0	2.496
13	0	3.599
14	0	4.742
15	0	5.986
16	1.449	0
17	2.909	0
18	4.463	0
19	5.818	0
20	7.875	0
21	1.324	4.010
22	3.359	6.717
23	7.148	0.497
24	4.236	3.039
25	5.194	1.679
26	2.733	1.263
27	5.166	2.158
28	6.408	3.345
29	0.734	3.888
30	5.579	4.971
31	7.212	4.174
32	5.491	6.444
33	2.533	2.177
34	5.816	7.754
35	1.314	0.734
36	3.577	6.034
37	6.705	3.878
38	2.680	5.179
39	2.608	1.773
40	1.507	2.128
41	0.759	7.519
42	5.127	8.613
43	1.949	3.242
44	2.477	6.335
45	3.821	7.992
46	0.985	8.218
47	4.371	1.166
48	1.592	0.871
49	4.627	1.925
50	5.026	7.882

Table S1. Concentration of Cha and Bif in calibration solutions of ethanol used to train a partial least squares regression (PLSR) model. Each solution was independently prepared.

Molecular Glass Eutectic temperatures:

Sample	T_{eu} / °C
Cur/Cli	89.0
Phl/Cli	87.7
Ros/Cli	88.6
Cha/Bif	116.7
Cha'/Bif	110.7
Cur/Bif	124.2
Tan/Nic	102.9

Table S2. Eutectic temperature (T_{eu}) of each eutectic mixture obtained using DSC (see **Figure S5-11** for a representative examples). The time taken to form the eutectic phase varies by sample, sample size and mixing efficiency. In general, the timescale is 10s of minutes to hours. For example, producing a homogenous sample of 10 g Cha/Bif requires approx. 40 min at stirring rate of 100 rpm (see **Supporting Video 1** for a timelapse).

Viscosity-temperature Parameters:

Sample	T / °C	η_0 / -	B / -	T_{VF} / -
Cur/Cli	140-50	4.59×10^{-6}	1288	-15.8
Phl/Cli	100-40	1.19×10^{-5}	1080	-11.6
Ros/Cli	100-40	4.91×10^{-6}	1197	-26.5
Cha/Bif	140-50	2.25×10^{-5}	998	2.40
Cha'/Bif	140-50	1.20×10^{-5}	1126	0.20
Cur/Bif	150-70	8.56×10^{-7}	1610	4.40
Tan/Nic	-	-	-	-

Table S3. Temperatures (T) probed for rotational viscosity-temperature experiments. Samples were isothermally conditioned at the upper temperature bound for 5 minutes in all cases. Temperature ramp rate, velocity and sampling interval were 2.0 $°C\cdot min^{-1}$, 1.0 $rad\cdot s^{-1}$ and 10 $s\cdot pt^{-1}$ for all experiments. Experimental data was fit to the VFT equation (**Equation 2**) using the parameters η_0, B, and T_{VF}. All r^2 values were > 0.999.

Amplitude Sweep Parameters:

Sample	T / °C	ω / $rad\cdot s^{-1}$
Cur/Cli	40	10
Phl/Cli	30	10
Ros/Cli	25	10
Cha/Bif	50	5
Cha'/Bif	50	10
Cur/Bif	75	5
Tan/Nic	-	-

Table S4. Temperature (T) and angular frequency (ω) at which amplitude sweep experiments were performed. Samples were isothermally conditioned for 5 minutes before measurement in all cases. Displacement was between 2×10^{-8} – 10.0 rad in all cases with 10 $pt\cdot dec^{-1}$ sampling rate.

Frequency sweep parameters:

Sample	T, ΔT/ °C	ΔT / °C	Δx / rad
Cur/Cli	30-120,	10	5×10^{-2}
Phl/Cli	30-100	10	2×10^{-3}
Ros/Cli	30-100	10	2×10^{-3}
Cha/Bif	25-50	5	1×10^{-4}
	50-120	10	1×10^{-4}
Cha'/Bif	40-80	10	1×10^{-4}
	35-40	1	1×10^{-4}
Cur/Bif	50-150	5	1×10^{-4}
Tan/Nic	-	-	-

Table S5. Temperature range (T), step size (ΔT) and displacement (Δx) at which frequency sweep experiments were performed. Samples were isothermally conditioned for 5 minutes before measurement in all cases.

Oscillatory viscosity-temperature sweep parameters:

Sample	T / °C	Δx / rad
Cur/Cli	20-100	1×10^{-2}
Phl/Cli	10-100	1×10^{-2}
Ros/Cli	50-120	2×10^{-3}
Cha/Bif	10-100	1×10^{-2}
Cha'/Bif	20-100	1×10^{-2}
Cur/Bif	50-150	1×10^{-4}
Tan/Nic	-	-

Table S6. Temperature range (T) and displacement (Δx) at which oscillatory viscosity-temperature experiments were performed. Angular frequency (ω) and temperature ramp rate were constant across all measurements at 10.0 rad·s^{-1} and 2 °C·min^{-1} respectively. All samples were isothermally conditioned for 5 minutes before measurement.

Cha Bif formation 116 C 45 min in 10 sec .mp4
This file cannot be rendered in this PDF. Please download the source file.

Filmonwater.mp4
This file cannot be rendered in this PDF. Please download the source file.
